# TFAP2A orchestrates gene regulatory networks and tubular architecture in kidney outer medullary collecting ducts

**DOI:** 10.1172/jci.insight.192361

**Published:** 2025-08-28

**Authors:** Janna Leiz, Karen I. López-Cayuqueo, Shuang Cao, Louisa M.S. Gerhardt, Christian Hinze, Kai M. Schmidt-Ott

**Affiliations:** 1 Department of Nephrology and Hypertension, Hannover Medical School (MHH), Hannover, Germany.; 2 Molecular and Translational Kidney Research, Max Delbrück Center for Molecular Medicine in the Helmholtz Association, Berlin, Germany.; 3 Department of Nephrology and Medical Intensive Care, Charite′ – Universitätsmedizin Berlin, Corporate Member of Freie Universität Berlin and Humboldt-Universität zu Berlin, Berlin, Germany.; 4 Fifth Department of Medicine, Faculty of Medicine Mannheim of the University of Heidelberg, University Medical Center Mannheim, Mannheim, Germany.

**Keywords:** Cell biology, Nephrology, Transcription

## Abstract

Mutations in the transcription factor TFAP2A are linked to congenital anomalies of the kidney and urinary tract in humans. While *Tfap2a* knockout (KO) in mouse collecting ducts leads to tubular epithelial abnormalities, its precise molecular functions in kidney tubules remain unclear. To investigate *Tfap2a*-dependent gene regulatory networks in the mouse kidney collecting ducts, we employed conditional KO (*Hoxb7-Cre; Tfap2a^fl/fl^*) models combined with transcriptomics. Histomorphological and physiological assessments of *Tfap2a*-KO mice revealed progressive postnatal dilation of the outer medullary collecting ducts. Integrating bulk and single-nucleus RNA sequencing with in silico motif mapping in ATAC-seq datasets demonstrated that *Tfap2a* is highly expressed and active in normal collecting duct principal cells. Comparative transcriptomics between 3-month-old *Tfap2a*-KO and control mice identified dysregulated genes associated with cell adhesion and WNT signaling, including *Alcam* and *Wnt9b*. These changes were confirmed by in situ hybridization. Our findings reveal that *Tfap2a* regulates medullary collecting duct diameter by orchestrating a transcriptional network involving *Wnt9b* and *Alcam*, providing insights into its role in kidney structural integrity.

## Abstract 2

Tfap2a regulates kidney collecting duct structure and function by controlling genes involved in cell adhesion and WNT signaling in mice.

## Introduction

Collecting ducts (CDs) comprise the most distal parts of renal tubules and coordinate key physiological processes, such as water homeostasis and extracellular fluid volume, electrolyte balance, blood pressure, and acid-base regulation (1, 2). During nephrogenesis, the mature CD system arises by repeated branching of the ureteric bud, a derivative of the nephric duct (3, 4). CD maturation and differentiation of its progenitor cell populations are strictly controlled by complex gene networks and crosstalk of regulatory pathways such as BMP, TGF-β, and WNT signaling (4–7). Dysregulation of these pathways during renal morphogenesis gives rise to congenital anomalies of the kidney and urinary tract (CAKUT) (8, 9). Manifestations of CAKUT comprise a large spectrum of renal anomalies and represent a frequent cause of chronic kidney disease in children and young adults (10–12).

Genetic studies in mice and humans identified a number of CAKUT-associated genes, many of them encoding transcription factors (8, 13). One of these genes encodes the transcriptional regulator TFAP2A, a member of the AP-2 transcription factor family (14, 15). This family consists of 5 members with partially overlapping functions, 2 of which, TFAP2A and TFAP2B, are expressed in the kidney (16–18). Tfap2a serves as a critical regulator of terminal distal nephron precursor differentiation in zebrafish (19), and it has been proposed as essential for the initiation of urinary tract and CD development in mice (20, 21). In humans, heterozygous missense mutations of TFAP2A lead to branchio-oculo-facial syndrome (BOFS), which is associated with renal malformations in approximately 35% of patients (22, 23). BOFS-associated malformations include renal dysplasia, renal agenesis, multicystic kidneys, and vesicoureteral reflux (14, 23).

Previous studies have indicated high expression levels of *Tfap2a* in developing and adult CDs (18, 19, 21, 24–27). Knockout of *Tfap2a* in the mouse CD causes widened epithelial tubules in the outer medulla (24). However, the detailed gene regulatory networks mediating the functions of Tfap2a in kidney epithelia and their potential roles in renal malformations are unknown. In this study, we induced CD-specific knockout of *Tfap2a* in mice and employed single-cell and bulk transcriptomics, bioinformatic integration with existing ATAC-seq datasets, in silico ChIP-seq, histomorphological phenotyping, and in situ molecular validation, to define the activity domains, target genes, and molecular functions of *Tfap2a* in the kidney.

## Results

### TFAP2A is the dominant AP-2 transcription factor in cells of the kidney’s medullary CD.

**Figure 1:**
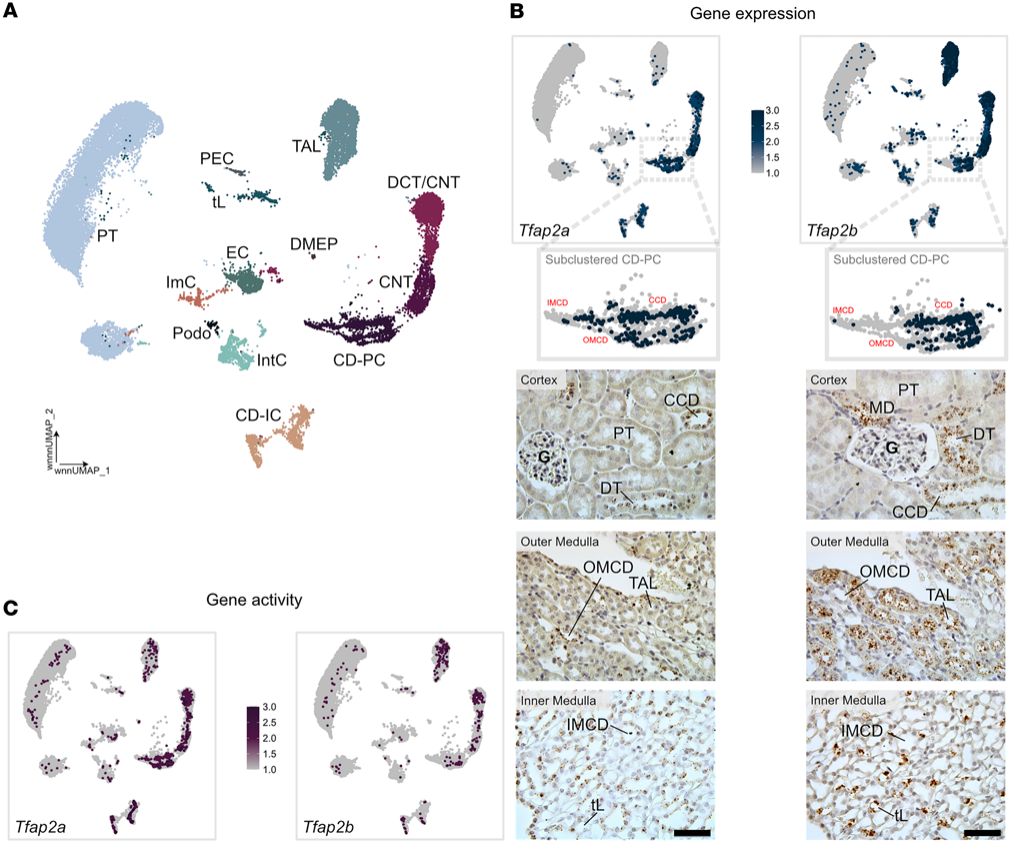
Transcription factors *Tfap2a* and *Tfap2b* show partly overlapping gene expression and gene activity in the adult kidney. (**A**) Uniform manifold approximation and projection (UMAP) embedding of multiomic sequencing data for mouse kidneys based on both RNA and ATAC data using a weighted nearest neighbor (wnn) analysis (27,802 nuclei, *n* = 3). Nuclei were annotated to podocytes (Podo), parietal epithelial cells (PEC), proximal tubule (PT) cells, thin limb (tL) cells, thick ascending limb (TAL) cells, distal convoluted tubule (DCT) cells, connecting tubule (CNT) cells, collecting duct principal cells (CD-PCs), collecting duct intercalated cells (CD-ICs), endothelial cells (ECs), interstitial cells (IntCs), and immune cells (ImCs) using known marker genes. (**B**) Gene expression domains of *Tfap2a* and *Tfap2b* in the kidney. Top: UMAP displaying expression domains of *Tfap2a* and *Tfap2b* mRNA in mouse kidneys. The color gradient ranges from gray (no expression) to dark blue (highest expression). CD-PCs were extracted and reclustered to identify subpopulations. Marker gene analysis enabled annotation as cortical CD (CCD), outer medullary CD (OMCD), or inner medullary CD (IMCD), which were then remapped onto the original UMAP to visualize their distribution within the global dataset. Bottom: In situ hybridization (RNAscope) for *Tfap2a* (left) and *Tfap2b* (right) mRNA (brown dots) in adult mouse kidney tissue. Expression was detected in the TAL, tL, macula densa (MD), distal tubule (DT; comprised of DCT and CNT), CCD, OMCD, and IMCD. Scale bars: 50 μm. (**C**) Chromatin accessibility of the *Tfap2a* and *Tfap2b* genes in cells of the kidney. Same UMAP as in **B**, displaying chromatin accessibility associated with the *Tfap2a* and *Tfap2b* genes as a color gradient ranging from gray (no accessibility) to dark purple (high accessibility).

Transcription factors regulate chromatin accessibility and gene expression to determine cellular identity and differentiation (26). *Tfap2a* is a member of the AP-2 family of transcription factors. A second member of this family, *Tfap2b*, has been identified as a critical regulator of distal nephron differentiation and its absence results in progressive distal convoluted tubule (DCT) abnormalities and β-catenin/mTOR hyperactivation that is associated with renal fibrosis and cyst formation (24). To compare expression and activity domains of *Tfap2a* and *Tfap2b* in the mouse kidney, we first performed a detailed assessment of *Tfap2a* and *Tfap2b* mRNA expression, chromatin accessibility, and transcription factor activity by reanalyzing single-cell multiomic ATAC and gene expression data from adult mouse kidneys (28) and by performing RNAscope in situ hybridizations in WT C57BL/6 mouse kidneys (Figure 1, A–C). Single-cell multiomic analyses from 27,802 nuclei (Supplemental Figure 1, A–C; supplemental material available online with this article; https://doi.org/10.1172/jci.insight.192361DS1) rendered 13 broad kidney cell types based on the expression of marker genes (Figure 1A and Supplemental Figure 1D). Gene expression data (Figure 1B) and chromatin accessibility analyses of the *Tfap2a* and *Tfap2b* genes (Figure 1C and Supplemental Figure 2) indicated that the *Tfap2a* gene was expressed and associated with open chromatin in cells from the DCT, connecting tubule (CNT), CD principal cells (CD-PCs), and CD intercalated cells (CD-ICs). In contrast, *Tfap2b* was strongly expressed and associated with open chromatin in cells from the thick ascending limb (TAL), DCT, and CNT, but less in cells from CD-PCs and CD-ICs. In situ hybridization to detect *Tfap2a* and *Tfap2b* mRNAs in WT mouse kidney sections corroborated these expression patterns and revealed that *Tfap2a* mRNA was highly expressed in cells from outer medullary collecting ducts (OMCDs) and inner medullary collecting ducts (IMCDs), whereas *Tfap2b* expression was minimal in these CD segments (Figure 1B). This indicated that *Tfap2a* is the predominant AP-2 transcription factor in kidney MCDs.

**Figure 2:**
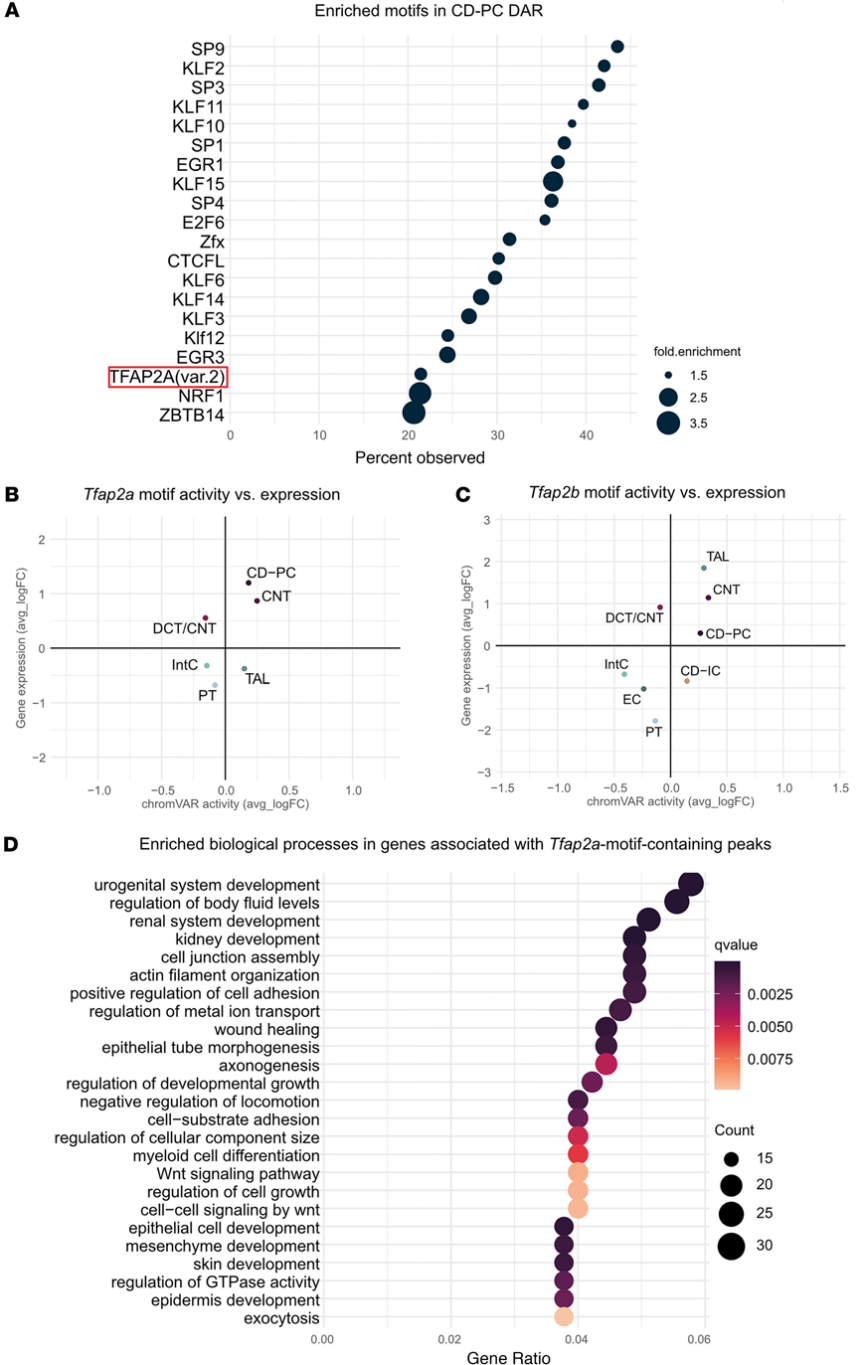
Transcription factor *Tfap2a* shows increased regulatory activity in collecting duct principal cells and is associated with pathways related to kidney development. (**A**) Motif enrichment analysis on the differentially accessible regions (DARs) in collecting duct principal cells (CD-PCs). The top 20 motifs with the highest observed frequency are shown (*P*_adj_ < 0.05). The Tfap2a motif MA0810.1 [TFAP2A(var.2)] is highlighted (red box). Dot size indicates the fold enrichment in comparison to the background dataset. (**B**) *Tfap2a* chromVAR motif activity plotted against Tfap2a gene expression. Significant differential chromVAR activity and transcription factor expression (determined by the Seurat FindMarkers function) were observed in the proximal tubule (PT), thick ascending limb (TAL), distal convoluted tubule (DCT), connecting tubule (CNT), collecting duct principal cells (CD-PCs), and interstitial cells (IntCs). (**C**) *Tfap2b* chromVAR motif activity plotted against *Tfap2b* gene expression. Significant differential chromVAR activity and transcription factor expression (determined by the Seurat FindMarkers function) were observed in the PT, TAL, DCT, CNT, CD-PCs, collecting duct intercalated cells (CD-ICs), endothelial cells (ECs), and IntCs. Cell types without significant activity or expression were not included in the plots (**B** and **C**). (**D**) Highly enriched biological processes identified for gene set of 546 genes associated with Tfap2a motif–containing peaks. Colors represent the *q* value for the depicted process; dot size the number of putative target genes associated with the process. The gene ratio (*x* axis) represents the number of putative target genes divided by the total number of genes associated with the respective pathway.

We next focused our analyses on CD-PCs by utilizing single-nucleus ATAC-seq data to identify CD-PC–specific “open” and “closed” chromatin regions. We identified 1,933 differentially accessible regions (DARs) of the genome (average log_2_[fold change] > 2, *P*_adj_ < 0.01) in CD-PCs when compared with all other kidney cell types. Among these DARs were regions associated with known CD marker genes (e.g., *Aqp2*, which encodes aquaporin-2) as well as regions associated with gene promoter and enhancer regions for *Tfap2a*, but not *Tfap2b* (Supplemental Table 1). We then used an unbiased approach (Signac) to identify enriched transcription factor motifs within these CD-PC–specific DARs, which yielded 67 enriched transcription factor motifs (*P*_adj_ < 0.05, log_2_[fold change] > 1.5). Notably, the TFAP2A(var.2) motif (MA0810.1) was among the top 20 enriched motifs when sorted by motif abundance (Figure 2A and Supplemental Table 2). In addition, chromVAR motif activity analysis indicated that AP-2 transcription factor motifs, including the TFAP2A(var.2) motif, showed a significantly increased activity within CD-PCs compared with other cell types (Supplemental Table 3). In accordance, *Tfap2a* motif activity and gene expression were high in CD-PCs and CNTs (Figure 2B and Supplemental Table 4), whereas *Tfap2b* motif activity and gene expression were high in CNTs and TALs (Figure 2C and Supplemental Table 4).

To gain deeper insights into molecular mechanisms and regulatory networks regulated by *Tfap2a*, we filtered the identified CD-PC DARs for the presence of a *Tfap2a* motif and identified the associated genes. In total, 625 open chromatin regions that associated with 546 distinct genes contained at least one *Tfap2a* motif (Supplemental Table 5). Biological processes associated with these genes included cell junction assembly, epithelial morphogenesis, kidney development, Wnt signaling, and GTPase activity (Figure 2D and Supplemental Table 6), further supporting the notion that *Tfap2a* regulates critical molecular networks in CD-PCs.

### Tfap2a is required to maintain tubule structure of renal CDs.

**Figure 3:**
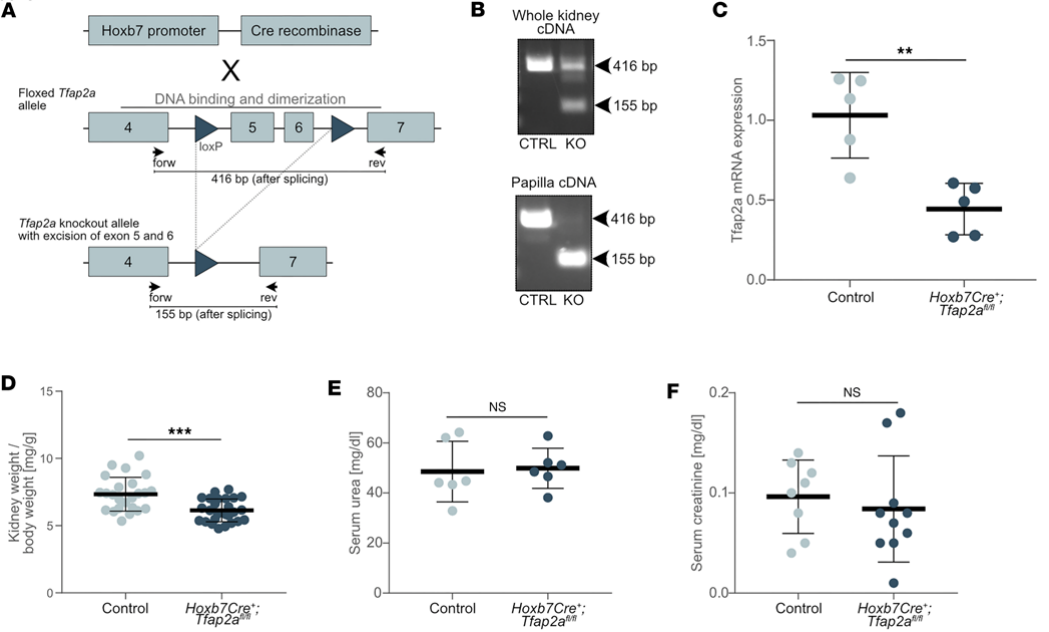
*Hoxb7Cre^+^;Tfap2a^fl/fl^* mice display reduced kidney weights but normal kidney function. (**A**) Breeding strategy to generate a collecting duct–specific knockout of *Tfap2a*. The *loxP* sites flank exons 5 and 6 encoding part of the DNA binding and dimerization domain, resulting in a loss of function after their excision. Primers (indicated by arrows) for knockout validation and respective product sizes after splicing are indicated below the alleles. (**B**) Knockout validation on cDNA from adult whole kidney and microdissected papillary tissue. Control animals (CTRL) displayed full-length alleles (416 bp), whereas *Hoxb7Cre^+^;Tfap2a^fl/fl^* (KO) mice showed shortened knockout alleles (115 bp). In whole kidney tissue, WT *Tfap2a* mRNA was still expressed, likely due to maintained expression in distal tubules. In papillary tissues, devoid of distal tubules, no residual WT *Tfap2a* mRNA was detected. (**C**) Expression levels of *Tfap2a* mRNA normalized to β-actin mRNA expression in whole kidney samples of newborn (P1–P2) control and *Hoxb7Cre^+^;Tfap2a^fl/fl^* mice. *Tfap2a* expression was downregulated by approximately 60%. (**D**) Kidney/body weight ratios of 3-month-old control and *Hoxb7Cre^+^;Tfap2a^fl/fl^* mice. (**E** and **F**) Serum urea and serum creatinine of 3-month-old control and *Hoxb7Cre^+^;Tfap2a^fl/fl^* mice. *n* ≥ 5 mice per group. Data are expressed as mean ± SD. Statistical significance was determined using a 2-tailed *t* test, without assuming a consistent SD. NS, not significant. **P < 0.01, ***P < 0.001.

We selectively deleted *Tfap2a* in the CD system by breeding *Tfap2a^fl/fl^* mice, which were engineered to carry *loxP* sites flanking exons 5 and 6 of the *Tfap2a* gene, with *Hoxb7Cre^+^* mice, which selectively induce Cre-mediated recombination in CDs and lower urinary tract epithelia (Figure 3A) (29). We confirmed that the resulting *Hoxb7Cre^+^;Tfap2a^fl/fl^* mice showed excision of exon 5 and 6 in whole kidney and in kidney papilla samples (Figure 3B) as well as reduced *Tfap2a* expression in whole kidney samples (Figure 3C).

*Hoxb7Cre^+^;Tfap2a^fl/fl^* mice were found to survive beyond weaning age at rates slightly lower than the expected Mendelian prediction (42%; 175 out of 416 animals; *P* = 0.0012 by χ^2^ test). Surviving adult *Hoxb7Cre^+^;Tfap2a^fl/fl^* mice were viable and fertile, with normal body weights when compared to control littermates. Kidneys of *Hoxb7Cre^+^;Tfap2a^fl/fl^* mice appeared smaller than those of littermate controls and showed mildly reduced kidney weight/body weight ratios (Figure 3D and Supplemental Figure 3).

Three-month-old *Hoxb7Cre^+^;Tfap2a^fl/fl^* mice exhibited no signs of reduced kidney function according to serum urea (Figure 3E) and serum creatinine levels (Figure 3F). Blood acid base analyses, urinary electrolyte concentrations, urinary osmolality, total daily urinary excretion, drinking volume, and body weights showed no differences between 3-month-old *Hoxb7Cre^+^;Tfap2a^fl/fl^* mice and control littermates (Supplemental Tables 7–9). When challenged with 24-hour water deprivation, *Hoxb7Cre^+^;Tfap2a^fl/fl^* mice displayed an ability to concentrate urine similar to littermate controls, indicating that *Tfap2a* expression is not responsible for renal osmoregulation.

**Figure 4:**
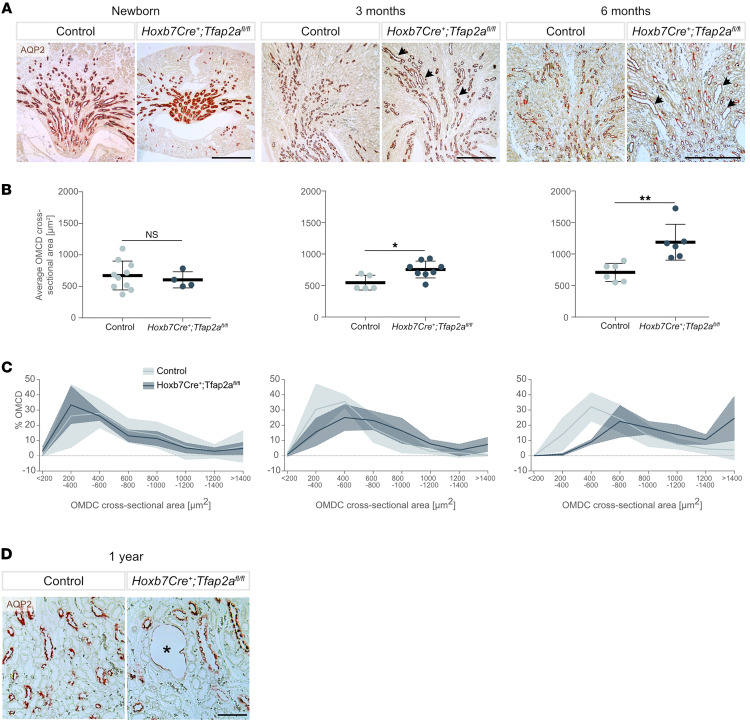
*Hoxb7Cre^+^;Tfap2a^fl/fl^* mice present dilated outer medullary collecting duct tubules in adult animals. (**A**) Immunohistochemistry for the collecting duct marker aquaporin-2 (AQP2) in newborn, 3-, and 6-month-old control and *Hoxb7Cre^+^;Tfap2a^fl/fl^* mice. Examples of dilated collecting duct tubules in adult mice are marked with dark arrow heads Scale bars: 500 μm. (**B**) Measurements of average outer medullary collecting duct (OMCD) cross-sectional areas for newborn, 3-, and 6-month-old control and *Hoxb7Cre^+^;Tfap2a^fl/fl^* mice. *n* ≥ 4 mice per group. Data are expressed as mean ± SD. Statistical significance was determined using a 2-tailed *t* test, without assuming a consistent SD. NS, not significant. *P < 0.05, **P < 0.01. (**C**) Percentage of OMCDs within a given range of cross-sectional area for the same newborn, 3-, and 6-month-old control and *Hoxb7Cre^+^;Tfap2a^fl/fl^* mice as in **B**. Solid lines represent means, and shadowed areas the respective SD. (**D**) Immunohistochemistry for the collecting duct marker AQP2 in aged (1-year-old) control and *Hoxb7Cre^+^;Tfap2a^fl/fl^* animals. An example of a dilated collecting duct tubule with flattened epithelium is marked with an asterisk. Scale bar: 100 μm.

We next examined the morphology of kidneys at different ages. In newborn mice, overall renal morphology was intact, but in adult animals a tubular dilation of MCDs was observed (Figure 4A). Measurements of cross-sectional areas, representing the total CD lumen, and of tubular diameters indicated a progressive tubular dilation (Figure 4, B and C, and Supplemental Figure 4). In aged animals (1 year old), some dilated tubules showed flattened epithelia (Figure 4D). These findings were consistent with the previously reported phenotype of *Aqp2Cre^+^Tfap2a^fl/fl^* mice (24).

### Tfap2a is not required to establish CD cell type abundances.

**Figure 5:**
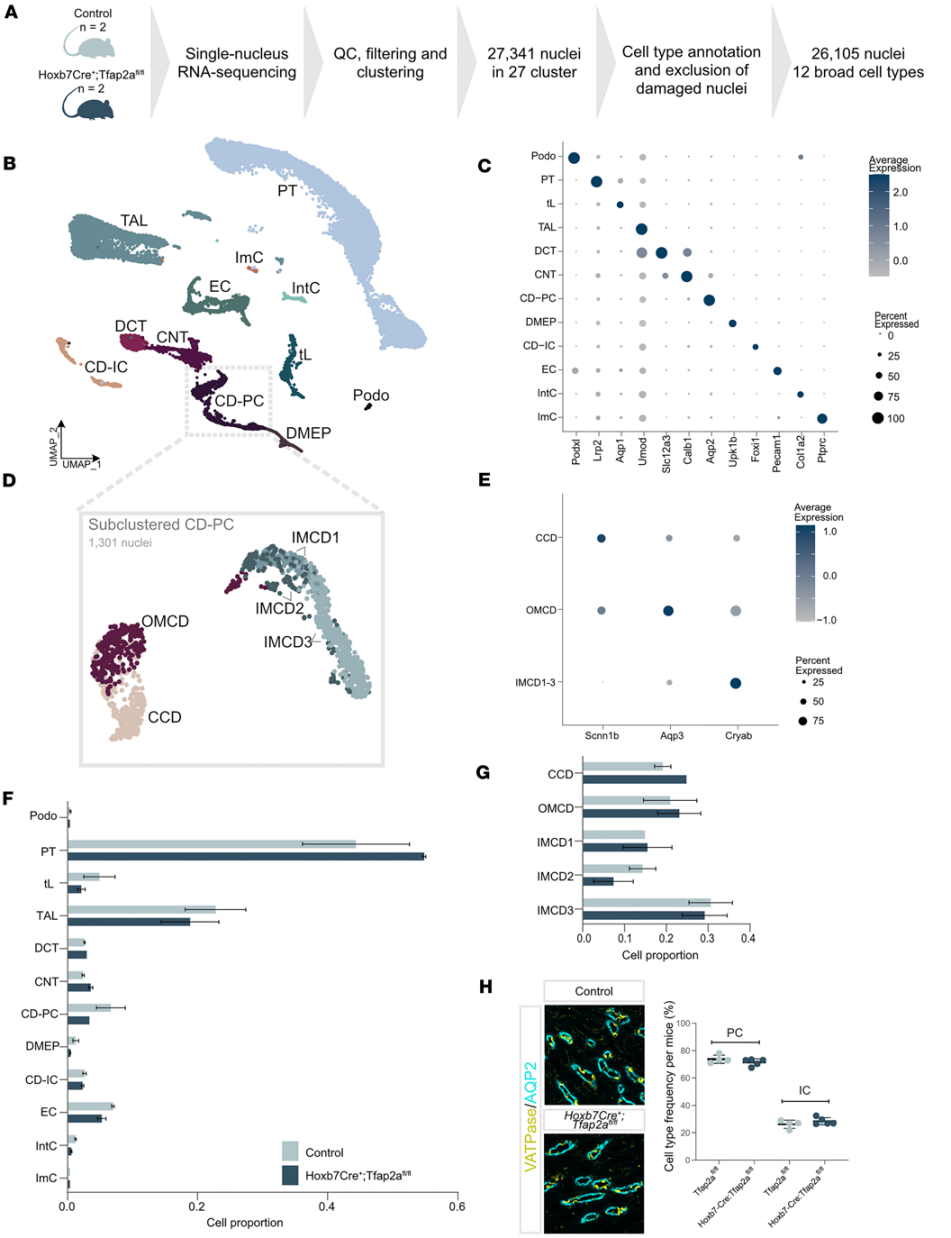
*Hoxb7Cre^+^;Tfap2a^fl/fl^* mice show no overt changes in cell type abundance compared to controls, based on snRNA-seq and immunofluorescence. (**A**) snRNA-seq was performed on kidneys from 3-month-old male control and *Hoxb7Cre^+^;Tfap2a^fl/fl^* mice (*n* = 2 per group). (**B**) Uniform manifold approximation and projection (UMAP) of snRNA-seq data (26,105 nuclei) from both genotypes. Nuclei were annotated using known marker genes as podocytes (Podo), proximal tubule (PT), thin limb (tL), thick ascending limb (TAL), distal convoluted tubule (DCT), connecting tubule (CNT), collecting duct principal cells (CD-PCs), collecting duct intercalated cells (CD-ICs), deep medullary epithelium of pelvis (DMEP), endothelial cells (ECs), interstitial cells (IntCs), and immune cells (ImCs). Damaged nuclei were excluded. (**C**) Dot plot showing expression of cell-type-specific markers in broad cell types. Dot color reflects scaled average expression; size indicates the percentage of cells expressing the gene. (**D**) UMAP of subclustered CD-PC nuclei, annotated into cortical (CCD), outer medullary (OMCD), and inner medullary (IMCD1–3) PCs. (**E**) Dot plot showing marker gene expression for CD-PC subclusters. (**F**) Cell type proportions across broad populations in both genotypes (mean ± SD; *n* = 2 per group). (**G**) Distribution of CD-PC subclusters in control and mutant mice (mean ± SD; *n* = 2 per group). (**H**) Left: Immunofluorescent staining for PC marker aquaporin-2 (AQP2, cyan) and intercalated cell marker V-ATPase B1/2 (yellow) in OMCDs from 11-week-old control and mutant mice. Scale bar: 100 μm. Right: Quantification of PC and IC percentages in OMCDs (*n* ≥ 4 mice per group; mean ± SD). No significant differences were observed (2-tailed *t* test, equal variances not assumed).

To understand the molecular basis of the abnormalities of CD architecture observed in *Tfap2a* deficiency, we performed single-cell-resolved transcriptome analyses in *Hoxb7Cre^+^;Tfap2a^fl/fl^* and littermate control mice. We obtained kidneys from 3-month-old *Hoxb7Cre^+^;Tfap2a^fl/fl^* and littermate control mice and processed them for single-nucleus RNA-seq (snRNA-seq) according to our established protocol (30). We chose this time point for analysis, as tubular dilation became evident at this stage but was not yet severe. We obtained transcriptomes from more than 25,000 nuclei with a median of 2,628 unique molecular identifiers (UMIs), 1,577 genes, and 0.97% mitochondrial reads per nucleus (Supplemental Figure 5, A and B, and Supplemental Table 10). There were no overt differences in median UMI, gene, or mitochondrial RNA count between *Hoxb7Cre^+^;Tfap2a^fl/fl^* and control mice (Supplemental Figure 5, C–E, and Supplemental Table 10). Clusters were summarized as 12 broad cell types representing a total of 26,105 nuclei (Figure 5A). Broad cell types included known renal epithelial cell types as well as immune, interstitial, and endothelial cells (Figure 5B). All cell types were assigned based on expression of known marker genes (Figure 5C). In *Hoxb7Cre^+^;Tfap2a^fl/fl^* mice, tubular dilation was observed in OMCDs but not CCDs. Therefore, we further subclustered CD-PCs to refine downstream analyses to the specific cortico-medullary segment, revealing 5 discrete CD-PC populations representing CCDs, OMCDs, and 3 subtypes of IMCD (subtypes 1–3) (Figure 5D), all of which were confirmed by marker gene expression (Figure 5E). Overall cell type abundances of broad and subclustered cell types were similar between *Hoxb7Cre^+^;Tfap2a^fl/fl^* and control mice (Figure 5, F and G, and Supplemental Table 10), suggesting that *Tfap2a* does not control nephron and CD segmentation or distribution of distinct PC populations in the kidney. In addition, we stained kidney sections of *Hoxb7Cre^+^;Tfap2a^fl/fl^* and control mice for the PC marker AQP2 and the IC marker V-ATPase. We found no differences in PC and IC abundance between *Hoxb7Cre^+^;Tfap2a^fl/fl^* and control mice (Figure 5H and Supplemental Table 10).

### Tfap2a controls signaling pathways involved in tubule formation and diameter maintenance.

**Figure 6:**
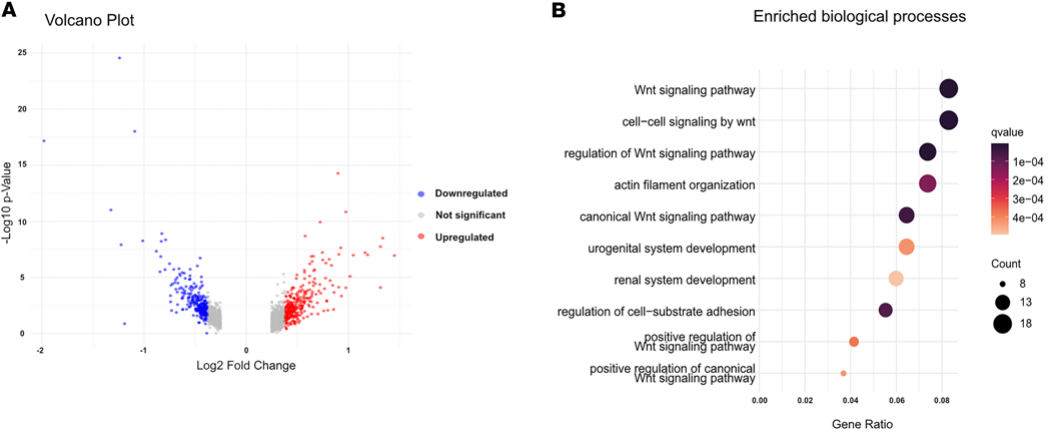
Transcriptomic changes and enriched biological processes in *Tfap2a*-deficient OMCD cells revealed by snRNA-seq. (**A**) Differentially expressed genes in outer medullary collecting duct (OMCD) cells. *P* < 0.05 by Wilcoxon’s rank sum test. (**B**) Highly enriched biological processes identified for 241 genes downregulated in *Tfap2a*-deficient OMCD cells. Colors represent the *q* value for the depicted process; dot size the number of putative target genes associated with the process. The gene ratio (*x* axis) represents the number of putative target genes divided by the total number of genes associated with the respective pathway.

To understand molecular pathways relevant to the phenotype observed in *Hoxb7Cre^+^;Tfap2a^fl/fl^* mice, we performed differential gene expression analysis in *Tfap2a*-deficient versus control OMCD cells based on snRNA-seq data. In total, 241 genes displayed decreased and 260 genes increased expression in *Tfap2a*-deficient compared with control conditions (*P* < 0.05, log_2_[fold change] ≥ 1.3; Figure 6A and Supplemental Table 11). Gene ontology analysis revealed that genes downregulated in *Tfap2a*-deficient OMCD cells were strongly associated with the same biological processes identified as likely *Tfap2a* target pathways in our initial analysis. Enriched biological processes included Wnt signaling, cell adhesion, and kidney development (Figure 6B and Supplemental Table 12). A number of differentially expressed genes, partially overlapping with those deregulated in OMCDs, was also found in IMCD1, IMCD2, and IMCD3, when comparing *Tfap2a*-deficient versus control mice (Supplemental Table 11). Nevertheless, we decided to focus our analysis on OMCDs in further downstream analyses.

Using Signac, we performed motif enrichment analysis on the multiomic dataset. Open chromatin regions associated with genes with decreased expression in *Tfap2a*-deficient OMCDs revealed an overrepresentation of *Tfap2a* and other AP-2 family member motifs (Supplemental Figure 6A). In contrast, upregulated genes showed no such enrichment (Supplemental Figure 6B). Overall, this is consistent with Tfap2a acting as a transcriptional activator rather than a repressor.

We performed an in silico ChIP-seq experiment on the multiomic dataset by filtering for open chromatin regions associated with the 241 genes with decreased expression in *Tfap2a*-deficient OMCDs and the presence of at least one *Tfap2a* motif in the respective DNA sequence. This approach resulted in 218 individual genes associated with 1,386 peaks, representing potential primary target genes of Tfap2a (Supplemental Table 13).

In addition to snRNA-seq, we performed bulk RNA-seq on whole kidney samples dissected from 10- to 12-week-old *Hoxb7Cre^+^;Tfap2a^fl/fl^* and control mice. In total, 360 genes were deregulated in *Tfap2a*-deficient compared with control kidneys (*P* < 0.05, log_2_[fold change] ≥ 1.3; Supplemental Table 14). Again, enriched pathways were associated with Wnt signaling and renal development (Supplemental Table 15). Highly enriched biological processes commonly identified for downregulated genes in snRNA-seq and bulk RNA-seq datasets could be summarized as 5 overarching groups: adhesion, epithelial and tube morphogenesis, tissue migration, kidney development, and Wnt signaling; further emphasizing an essential regulatory role for *Tfap2a* in these processes.

### Tfap2a deletion leads to Wnt9b and Alcam downregulation.

Apical-basal polarity and WNT/planar cell polarity are both essential for tubulogenesis and epithelial differentiation (31–33). Two genes associated with cell adhesion and Wnt signaling were deregulated in *Tfap2a*-deficient mice: Alcam (34, 35), a member of the neuronal immunoglobulin-like domain superfamily of cell-adhesion molecules, and Wnt9b, encoding a Wnt signaling factor.

Alcam is involved in lupus nephritis by activating T cells (36) and is crucial for proper nephrogenesis (37). During embryonic kidney development in *Xenopus laevis*, Alcam is regulated by a β-catenin–independent Wnt signaling pathway, specifically the Wnt/JNK/Alcam branch (37). Wnt9b acts via the planar cell polarity (PCP) pathway and its inactivation in mice results in CD cyst formation (38).

**Figure 7:**
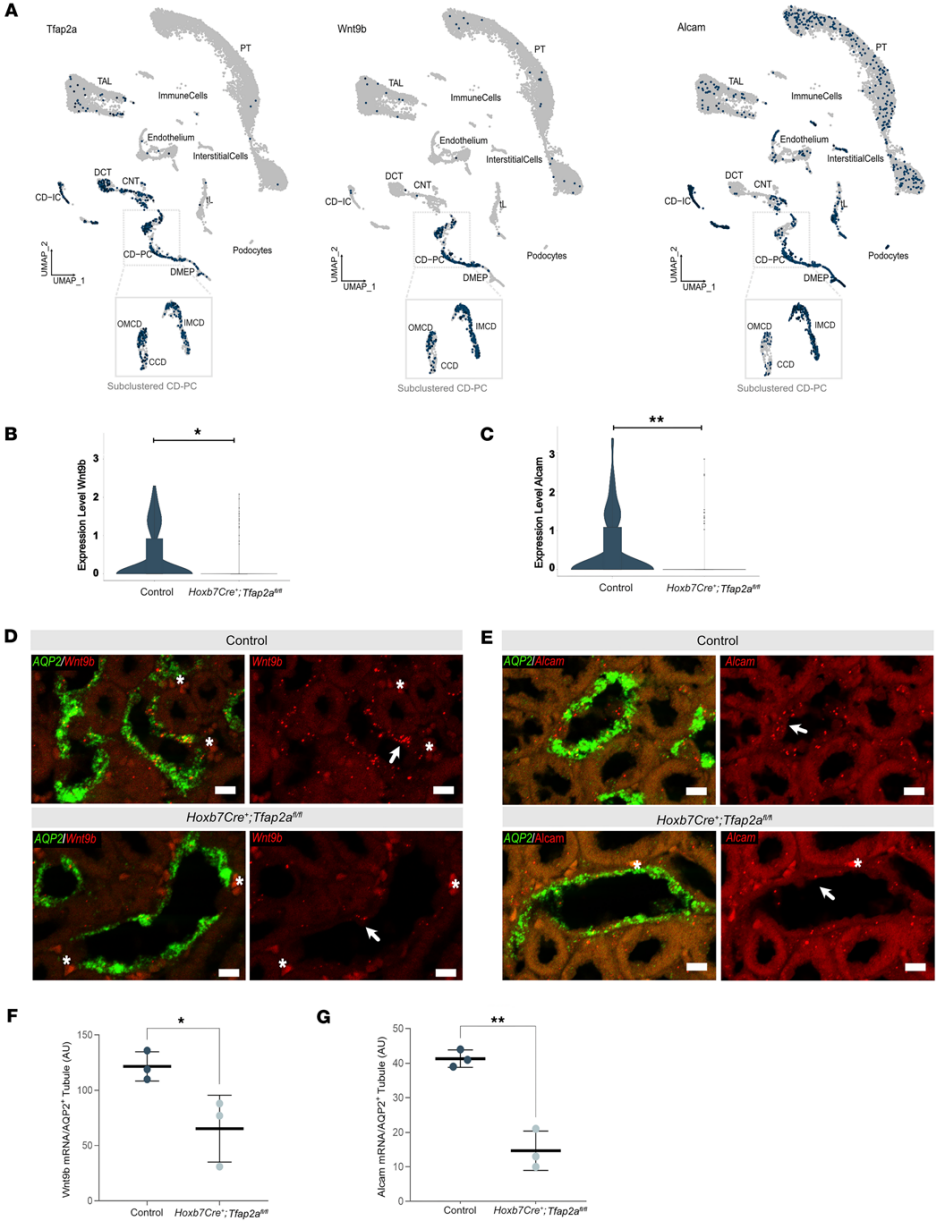
*Tfap2a* regulates Wnt signaling and cell adhesion. (**A**) Feature plot displaying the expression domains of *Tfap2a* mRNA, *Wnt9b* mRNA, and *Alcam* mRNA in broad cell types and subclusters of collecting duct principal cells (CD-PCs) in 3-month-old control mice (*n* = 2). The color gradient for feature plots ranges from gray (no expression) to blue (highest expression). PT, proximal tubule; TL, thin limb; TAL, thick ascending limb; DCT, distal convoluted tubule; CNT, connecting tubule; CD-IC, collecting duct intercalated cells; DMEP, deep medullary epithelium of the pelvis; CCD, cortical collecting duct; OMCD, outer medulla cortical collecting duct; IMCD, inner medulla cortical collecting duct. (**B**) Downregulated expression of *Wnt9b* and (**C**) *Alcam* mRNA in OMCD-PCs (based on snRNA-seq) of 3-month-old *Hoxb7Cre^+^;Tfap2a^fl/fl^* mice (*n* = 2/genotype). Data assessed with Seurat’s FindMarkers function. (**D**) In situ hybridization (representative pictures) of *Wnt9b* and (**E**) *Alcam* (both in red), along with *Aqp2* mRNA (green), in the OMCDs of 3-month-old control and *Hoxb7Cre^+^;Tfap2a^fl/fl^* mice. White arrows indicate examples of *Wnt9b* and *Alcam* mRNA expression. Asterisks indicate nonspecific red blood cell staining. Scale bars: 10 μm. (**F**) *Wnt9b* and (**G**) *Alcam* mRNA quantification per *Aqp2* mRNA–positive tubule (only PCs were considered for analysis). Each data point represents an estimate of the average number of mRNA molecules per tubular cross section per mouse (indicated in arbitrary units, AU; *n* of 10 tubules were analyzed per mouse, *n* = 3 mice/genotype). Data are expressed as mean ± SEM. Statistical significance was determined using a 2-tailed *t* test. *P < 0.05, **P < 0.01.

We examined *Alcam* and *Wnt9b* mRNA expression and its overlap with *Tfap2a* mRNA expression in our snRNA-seq dataset. *Alcam* mRNA was highly abundant in the CD (in both PCs and ICs) and detectable in additional cell types, while *Wnt9b* mRNA expression was specific to CD-PCs (Figure 7A). *Alcam* and *Wnt9b* mRNA expression was reduced in OMCD-PCs of *Hoxb7Cre^+^;Tfap2a^fl/fl^* mice compared with control mice (Figure 7, B and C). RNAscope in situ hybridizations validated reduced *Wnt9b* and *Alcam* mRNA expression in OMCDs of *Tfap2a*-deficient mice (Figure 7, D–G).

## Discussion

Our study indicates that *Tfap2a* is a critical factor that regulates and maintains the postnatal development and epithelial architecture of renal CDs and does so by regulating genes involved in epithelial cell differentiation, including WNT regulators and cell adhesion molecules. *Tfap2a*-deficient CDs displayed progressive postnatal tubular dilation that was accompanied by dysregulation of genes associated with Wnt signaling pathways. β-Catenin–dependent and –independent Wnt pathways are important for kidney development. Wnt signaling is known to be required for proper nephrogenesis and is downregulated in postnatal kidneys, but an ongoing, lower expression of Wnt components in adult kidneys suggests that Wnt activity is relevant even after nephrogenesis has ceased (39–41). A number of studies has demonstrated consequences of Wnt deregulation and its role in CAKUT, cystogenesis, and chronic kidney disease (42).

One of the target genes of *Tfap2a* we identified is *Wnt9b*, a well-characterized WNT that activates both canonical and non-canonical signaling pathways (38). Previous studies indicated that *Wnt9b* maintains tubular diameters in postnatal kidneys via the non-canonical PCP pathway. Knockout of *Wnt9b* results in progressive tubule dilation and cyst formation (38), partly resembling the *Tfap2a* phenotype described herein. In fact, a recent study, utilizing RNA-, ATAC-, and H3K4me3 ChIP-seq, identified *Wnt9b* as a likely Tfap2a target gene in the craniofacial ectoderm (43). Development of the craniofacial ectoderm is highly dependent on Tfap2a and a craniofacial component has been described in BOFS (14, 44, 45). PCP describes the harmonized orientation of cells in a tissue plane and coordinates convergent extension and oriented cell division during tubule formation (46–49). Oriented cell division is only established around birth and thus might explain overall intact renal morphology in newborn mice and the postnatal onset phenotype observed in our model (47). However, cell division rates significantly decrease in adult animals and in a model of polycystic kidney disease, misorientation has even been found to follow tubular dilation (50). Therefore, defects in oriented cell division cannot fully explain the advancing tubular dilation observed in aged (1 year old) *Hoxb7Cre^+^;Tfap2a^fl/fl^* mice and other mechanisms are likely to contribute.

We also found altered expression of cell adhesion machinery components in *Tfap2a*-deficient CDs based on transcriptomic signatures. It is well established that PCP interacts with cell adhesion signaling (51). Thus, loss of proper PCP in *Hoxb7Cre^+^;Tfap2a^fl/fl^* might result in reduced cell adhesion leading to dilated tubules. We found the cell-adhesion molecule *Alcam* consistently downregulated in our datasets. Previous findings suggested Alcam to be involved in adherens junction formation (52, 53). In *Xenopus laevis*, Alcam is required for embryonic kidney development (37) and is a direct target gene of β-catenin–independent Wnt signaling. In humans, *ALCAM* was found to be involved in capillary tube formation (54).

Overall, our study suggests that *Tfap2a* controls a transcriptional circuitry that includes *Wnt9b* and *Alcam* as potential mediators of tubular architecture and lumen size regulation. Given the complex nature of Wnt/PCP signaling and its crosstalk with other pathways and cell components, the exact mechanisms of tubule dilation in *Tfap2a*-deficient CDs will require further evaluation. Different factors might contribute to lumen expansion in *Tfap2a*-deficient CDs. In addition to defective PCP and oriented cell division, cytoskeletal rearrangements and altered cell adhesion in *Tfap2a* deficiency might promote altered tubular architecture with consequential aberrant tubular flow, mediating a feedback loop reinforcing progressive CD dilation over time.

In sum, our study provides molecular and cellular insights into functions of *Tfap2a* in CD epithelia and uncovers molecular circuitry relevant for congenital kidney diseases. However, several limitations must be acknowledged. First, our motif enrichment analysis does not allow discrimination between TFAP2A- and TFAP2B-specific binding due to their nearly identical DNA binding motifs. Paralog-specific regulatory activity would require additional experiments such as ChIP-seq or CUT&RUN using paralog-specific antibodies or tagged alleles. Second, although our data indicate a prominent role for TFAP2A in the OMCD, we cannot exclude functional redundancy with TFAP2B — particularly in regions like the cortical CD where they are coexpressed — which may explain the relatively mild phenotype observed in the *Tfap2a* conditional knockout. Moreover, our analysis of open chromatin regions extended beyond promoter-proximal sites to include distal regulatory elements, which introduces interpretive complexity when assigning regulatory function. While multimodal integration improved the specificity of these associations, further mechanistic studies are required to validate direct gene regulatory interactions. Finally, our study lacks definitive mechanistic confirmation of TFAP2A-driven regulation of Wnt9b and Alcam, which should be addressed in future work using gene-specific perturbation and chromatin binding assays.

## Methods

### Sex as a biological variable.

Male and female animals were used in the experiments.

### Animals.

Kidneys were obtained from 8- to 12-week-old C57BL/6N male mice (Charles River, Germany) to determine the mRNA expression domains of *Tfap2a* and *Tfap2b*.

CD *Tfap2a*-knockout mice were generated by crossing mice with homozygous floxed *Tfap2a* alleles (*Tfap2a^fl/fl^*; The Jackson Laboratory, 023406) (55) and mice carrying a homeobox B7–driven Cre recombinase (*Hoxb7Cre^+^*) (29) gifted from Carlton Bates (Children’s Hospital of Pittsburgh). Resulting *Hoxb7Cre^+^;Tfap2a^fl/WT^* animals were again bred with *Tfap2a^fl/fl^* mice. To generate experimental animals, the resultant *Hoxb7Cre^+^;Tfap2a^fl/fl^* were bred with *Hoxb7Cre^–^;Tfap2a^fl/fl^* animals. Cre-negative littermates were used as controls in experiments. Animals were maintained on a 12-hour light/dark cycle and had ad libitum access to food and water.

### RNA extraction.

Total RNA was extracted from whole kidney samples using the RNeasy Mini Kit (Qiagen, 74104). For microdissected papillary samples, the RNeasy Micro Kit (Qiagen, 74004) was used.

For kidney tissue disruption, frozen samples were transferred to ceramic bead–filled tubes (Bertin Technologies, KT03961-1-102.BK) containing QIAazol lysis reagent (Qiagen, 79306) and homogenized using a Precellys 24 tissue homogenizer (Bertin Technologies).

### Bulk RNA-seq.

For bulk RNA-seq, total RNA was extracted and library construction and sequencing was performed by Novogene. Libraries were sequenced on Illumina NovaSeq 6000 flow cells (paired-end, 150 bp).

Provided FASTQ files were aligned using STAR and the mm9 genome, reads were counted using featureCounts with -p -t exon -O -g gene_id -s 0 (56). Sequencing quality was assessed using FASTQC (57).

### cDNA synthesis.

RNA concentration and integrity were evaluated with a NanoDrop Spectrophotometer (Thermo Fisher Scientific) and 2100 Bioanalyzer Instrument (Agilent Technologies).

For cDNA synthesis, the RevertAid First Strand cDNA Synthesis Kit (Thermo Fisher Scientific, K1622) was used with 500 ng of total RNA according to the manufacturer’s instructions.

### Quantification of Tfap2a knockdown.

Real-time quantitative PCR (qPCR) was performed using 1 μL of synthesized cDNA and the FastStart Universal SYBR Green Master (ROX) according to the manufacturer’s instructions (Roche, 4913914001). Primer sequences used were qPCR_Tfap2a_forw (CCTAGCCAGGGACTTTGGGTA) and qPCR_Tfap2a_rev2 (CATGGGAGATGAGGTTGAAGT).

The forward primer targets a sequence transcribed from exon 6. This exon is missing in TFAP2A-deficient CDs of knockout animals. Relative expression levels of target mRNA were normalized to β-actin (*Actb*) mRNA expression and calculated using 2^–ΔΔCt^ (58).

### Knockout validation.

cDNA from control and *Hoxb7Cre^+^;Tfap2a^fl/fl^* mice was used with the Phire Animal Tissue Direct PCR Kit. Primer sequences used were Tfap2a_4_forw (GTCACGGTGGCGGAAGTACA) and Tfap2a_7_rev (ATAGGATTGGGCCGCGAGTT).

Excision of exon 5 and 6 was demonstrated by a smaller PCR product.

### Sample collection.

*Hoxb7Cre^+^;Tfap2a^fl/fl^* mice and control littermates were sacrificed as newborn (P0–P2) or as adult animals of 3, 6, or over 12 months old. Body weight was determined, and immediately following, blood samples were collected from the heart and mixed with heparin (1 mg/mL) to avoid blood clots. The plasma was collected and kept at –80°C for further analysis.

Kidneys were harvested, washed in ice-cold PBS, and weighed. For staining procedures, the tissue was fixed in 4% paraformaldehyde and embedded in paraffin following standard protocols; for RNA isolation, whole kidneys were immediately placed in liquid nitrogen and kept at –80°C for further analysis.

### Metabolic cages.

For urine collection, 10-week-old *Hoxb7Cre^+^;Tfap2a^fl/fl^* and control mice were kept in metabolic cages for 24 hours under (i) baseline (water ad libitum) or (ii) thirsting conditions. Body weight was determined before and after the experiment. In baseline experiments, drinking volume (normalized to body weight) was measured over 24 hours. The collected urine was analyzed regarding the volume (urinary excretion over 24 hours, normalized to body weight) and its osmolality using a Single-Sample Freezing Point Osmometer (Gonotec).

### Blood gas and electrolyte analysis.

For blood gas analysis, 2- to 3-month-old *Hoxb7Cre^+^;Tfap2a^fl/fl^* and control mice were sacrificed and whole blood samples were collected in heparinized petri dishes. The blood was immediately analyzed using the iStat handheld blood analyser (Abbott) and iStat CG8+ cartridges (Abbott, 03P88-25).

Urine and blood plasma samples of 10-week-old *Hoxb7Cre^+^;Tfap2a^fl/fl^* and control mice were analyzed regarding their electrolyte concentrations. Measurements were performed by the Preclinical Research Center (PRC) of the Max Delbrück Center for Molecular Medicine (MDC).

### Immunohistochemistry and immunofluorescence.

Paraffin-embedded kidney sections (4 μm) were dewaxed and hydrated. For antigen retrieval, slides were submerged in 10 mM citrate buffer and boiled for 20 minutes before cooling down at room temperature. Endogenous peroxidase activity was blocked for 10 minutes with 3% hydrogen peroxide in immunohistochemistry assays. Samples were blocked with PBS containing 1% BSA and 0.5% Triton X-100 (blocking solution) for 1 hour at room temperature. The primary antibodies against AQP2 (Santa Cruz Biotechnology, sc-9882) or V-ATPase B1/2 (Santa Cruz Biotechnology, sc-20943) were diluted 1:200 in blocking solution and incubated for 1 hour at room temperature or overnight at 4°C.

For immunohistochemistry assays, the secondary anti-goat IgG (H+L)-peroxidase antibody (Sigma-Aldrich, SAB3700284) was diluted 1:500 in blocking solution and incubated for 1 hour at room temperature. The sections were incubated 10–15 minutes with High Sensitivity Substrate Chromogen (Dako, K3461) and then mounted using Kaiser′s glycerol gelatin-based mounting medium (Merck, 109242). Images were captured on a Leica DM2000 LED bright-field microscope.

For immunofluorescent staining, AffiniPure donkey anti-goat IgG (H+L) (Alexa Fluor 647, Jackson ImmunoResearch, 705-605-147; 1:300) and ReadyProbes donkey anti-rabbit IgG (H+L) (Alexa Fluor 488, Thermo Fisher Scientific, R37118; 1:1,000) secondary antibodies were diluted in blocking solution and incubated for 1 hour at room temperature. The sections were then incubated with DAPI (1 μg/mL) for 5 minutes before mounting with fluorescence mounting medium (Dako, S3023). Sections were analyzed using a Leica DMi8 fluorescence microscope.

### Cell counting.

For CD-PC and CD-IC counting, several non-overlapping images per animal (10–12 weeks) were acquired from areas in the cortical, outer medullary, and inner medullary segment. Images were opened in ImageJ version 1.52a and autoadjusted for color and contrast to enhance visibility (59). For counting, the Cell Counter plugin was used. AQP2- and V-ATPase–positive cells were counted for all CDs fully shown. All measurements were carried out in a blinded manner.

Percentages of both cell types were calculated for the respective area and averaged per animal. Statistical significance was determined using a 2-tailed *t* test, without assuming a consistent standard deviation (SD).

### Morphometric analysis.

Paraffin-embedded kidney slices were stained for AQP2 (Santa Cruz Biotechnology, sc-9882) and images were captured on a Leica DM2000 LED bright-field microscope. Images were opened in ImageJ (59). All measurements were carried out in a blinded manner. A scale bar was set for all images. For measurements of cross-sectional areas, the area of the outer medulla was circled using the freehand selection tool. The area was duplicated and transformed into a binary image. Using the elliptic tool and filler function, CD shapes were filled. The wand tool was used for measurements of cross-sectional areas of tubules approximating an even circle. To measure tubule diameter, straight lines were drawn perpendicular to the longitudinal axis of the CD. For each animal, the average tubule diameter, cross-sectional area, and respective SDs were calculated. In addition, proportions of tubules within a given size range (bins of 200 μm^2^ for cross-sectional areas and 5 μm for tubule diameter) were calculated for each animal.

### Single-nucleus multiomic sequencing – preprocessing and data analysis workflow.

The multiomic dataset was accessed via the NCBI Gene Expression Omnibus (GEO GSE209610). Preprocessed data were downloaded for GSM6380583, GSM6380584, GSM6380585, GSM6380586, GSM6380595, and GSM6380596, representing gene expression and ATAC libraries of control samples from Ki67^cre/ERT2^; INTACT mice.

The R packages Seurat (60) (version 4.3.0) and Signac (61) (version 1.9.0) were used to process the data. Initial filtering of the Seurat object containing the gene expression and ATAC-seq data included nuclei with genes/cell greater than 350 and less than 3,500; RNA count greater than 500 and less than 8,000; ATAC count greater than 1,000 and less than 100,000; percentage mitochondrial genes less than 1, percentage ribosomal genes less than 3, and transcriptional start site enrichment score greater than 2. Seurat objects of each sample were merged into one object. RNA data were normalized with the NormalizeData() function in Seurat and variable features identified with FindVariableFeatures(). Datasets were integrated using the SelectIntegrationFeatures(), FindIntegrationAnchors(), and IntegrateData() functions with dims = 1:30, and k.anchor = 5. ATAC data were normalized using the RunTFIDF () function in Signac.

On the basis of the principle component analysis (RunPCA()) of the RNA assay and a singular value decomposition analysis (RunSVD()) of the ATAC assay, a weighted combination of the RNA and the ATAC data was calculated using FindMultiModalNeighbors(). Dimensionality reduction and cluster identification were performed using RunUMAP(), and FindClusters() with resolution = 0.8. Clusters were assigned to renal cell types based on marker gene expression. Identified clusters representing the same cell type were summarized as one cluster (= broad cell type). Clusters containing multiplets (marker gene expression for several cell types) were excluded from further analysis. The final Seurat object contained 27,802 nuclei.

### Gene-specific chromatin accessibility, differential expression, differential accessibility, and motif activity.

Chromatin accessibility at loci encoding *Tfap2a* and *Tfap2b* was calculated and visualized using the GeneActivity() function (which includes the gene body and 2 kb upstream promoter region by default) and visualized with the CoveragePlot() function. Differentially expressed genes (Wilcoxon’s rank sum test, min.pct = 0.1, logfc.threshold = 0.25) and DARs (logistic regression, min.pct = 0.05, logfc.threshold = 0.25) in CD-PCs compared with other kidney cells were identified using the FindMarkers() function. An adjusted *P* value of less than 0.01 was considered statistically significant. FindMotifs() was used to identify enriched motifs in DAR of CD-PCs.

Transcription factor motif activity was calculated using chromVAR (62) (version 1.16.0) and the JASPAR2020 database (63). Differential transcription factor motif activity in CD-PCs compared to other kidney cells was determined using the FindMarkers() function with mean.fxn = rowMeans, fc.name = “avg_diff”, test.use = “LR”, and min.pct = 0.1; motif activity was filtered for an adjusted *P* value of less than 0.01.

To identify a potential *Tfap2a* target gene set, the DNA sequence of each CD-PC DAR (average log_2_[fold change] > 2) was scanned for the presence of *Tfap2a* motifs by using the CreateMotifMatrix() function and filtering for the presence of at least one Tfap2a motif (MA0810.1 == ‘TRUE’ | MA0872.1 == ‘TRUE’ | MA0003.4 == ‘TRUE’).

### snRNA-seq library preparation.

For snRNA-seq, kidneys of 12-week-old male *Hoxb7Cre^+^;Tfap2a^fl/fl^* or control mice (*n* = 2 per group) were dissected and washed in ice-cold PBS. A middle slice of 1–2 mm was taken, and the cortex was trimmed to enrich for medullary cell types. Mouse kidney specimens were stored in precooled RNAlater (Invitrogen, AM7020) at 4°C for 24 hours and then stored at –80°C until nuclei isolation as reported in Leiz et al. (30).

All samples were subjected to single-cell sequencing following the 10x Genomics protocol (no. CG000204 Rev D) for Chromium Next GEM Single Cell 3′ v3.1 chemistry targeting 9,000–10,000 nuclei. Obtained libraries were sequenced on Illumina HiSeq 4000 sequencers (paired-end). Digital expression matrices were generated using the 10x Genomics Cell Ranger version 3.0.2, with –force-cells 10000 against a genome composed of the mouse mm10.

### snRNA-seq – preprocessing and data analysis workflow.

Sequencing data generated from *Hoxb7Cre^+^;Tfap2a^fl/fl^* and control mice were analyzed with Seurat version 3.2.1 (64). Initial filtering was performed by excluding nuclei with genes/cell of less than 500 or greater than 4,000, and mitochondrial reads greater than 5%. Genes expressed in less than 3 nuclei were not included in the analysis.

Nuclei passing the initial quality control were analyzed following Seurat’s workflow for data integration (64) with default parameters for NormalizeData(x) and FindVariableFeatures(x), followed by RunUMAP (dims = 1:30), FindNeighbors (dims = 1:30), and FindClusters (resolution = 0.5). Clusters were assigned to renal cell types based on their marker gene expression. Identified clusters representing the same cell type were summarized to one cluster (broad cell type). Nuclei from clusters showing marker gene expression for more than one cell type or high percentage of mitochondrial reads were assigned as damaged and excluded (2 clusters with 1,236 nuclei). The final Seurat object contained 26,105 nuclei. Relative abundances were calculated for broad cell types. Replicates from one group were averaged.

### Subclustering of CD-PCs.

For subclustering of CD-PCs, all nuclei with this identity (*n* = 1,301) were used as a subset. Neighboring nuclei were clustered and visualized in a uniform manifold approximation and projection (UMAP) based on 30 dimensions, k.param = 5, a resolution of 0.2., and otherwise default settings. Resulting subcluster were annotated based on known marker genes for PC subtypes (27). Relative abundances were calculated for subclustered CD-PCs for both groups.

### Differential gene expression analysis of sn- and bulk RNA-seq data.

Differential gene expression analyses for broad cell types and subclustered CD-PC types were performed with Seurat’s FindMarkers function with a min.pct = 0.05 or min.pct = 0.01, respectively, logfc.threshold of 0.25, and test = wilcox.

Differential gene expression analysis of bulk RNA-seq data was performed using the DESeq2 package version 1.34.0 (65). Input to DESeq2 were the raw count matrices. For kidneys, the standard DESeq protocol was followed.

### Pathway enrichment analysis for putative target and differentially expressed genes.

Enriched pathways were analyzed using the enrichGO() function in the clusterProfiler package version 4.2.2 (66). Enriched biological pathways were determined separately for genes up- and downregulated in the respective dataset. Adjustments of *P* values were calculated using the false discovery rate. Terms with a *P*- and *q*-value cutoff of less than 0.05 were considered significant.

### In silico ChiP-seq workflow.

The getMatrixSet() function from the JASPAR2020 R package (63) and the CreateMotifMatrix() function from Signac (61) were used to determine the presence of transcription factor motifs in open chromatin regions of the multiomic dataset. For each peak, the associated gene was identified using the ClosestFeature() function. Features were filtered for genes deregulated in OMCD-PCs and the presence of at least one Tfap2a motif in their respective DNA sequence.

### RNAscope in situ hybridizations.

The RNAscope 2.5 HD reagent kit-brown (Advanced Cell Diagnostics [ACD], 322300) was used to perform chromogenic in situ hybridizations on formalin-fixed, paraffin-embedded kidney sections with probes directed against *Tfap2a* (ACD, 319101) and *Tfap2b* mRNA (ACD, 535151). The RNAscope multiplex fluorescent reagent kit v2 (ACD, 323100) was used to perform fluorescent in situ hybridizations on formalin-fixed, paraffin-embedded kidney sections with probes directed against *Alcam* (ACD, 462061), *Wnt9b* (ACD, 405091), and *Aqp2* (ACD, 452411) mRNAs. Images of the hybridized sections were captured on a Leica DM2000 LED bright-field microscope or Zeiss LSM 980 confocal scanning microscope. The expression levels of *Alcam* and *Wnt9b* mRNAs in *Aqp2* mRNA–positive tubules (PCs) were quantified using ImageJ (59). To quantify RNA expression, the average area of individual RNAscope dots, representing single RNA molecules, was first determined for both *Alcam* and *Wnt9b* (6 different pictures from 3 WT mice). Subsequently, the total positive area for *Alcam* or *Wnt9b* within *Aqp2*-positive OMCDs was measured for each section (nonoverlapping pictures of 10 different collecting ducts per mouse). This total positive area was then divided by the previously calculated average area of a single dot, providing an estimate of the total number of RNA molecules present per OMCD.

### Statistics.

All RNA-seq data were analyzed using R (67), while all other data were processed with GraphPad Prism. Statistical tests, along with sample sizes (*n*), are specified in the corresponding figure legends. A *P* value of less than 0.05 was considered significant.

### Study approval.

All animal experiments were approved by and performed in compliance with local authorities (LAGeSo Berlin, Germany).

### Data availability.

snRNA-seq and bulk RNA-seq data have been deposited to the NCBI GEO, under accession numbers GSE290794 and GSE282959. All data supporting this research are included in the manuscript (supplemental tables and Supporting Data Values file). All codes used in this manuscript are available at https://github.com/KLopezCay/TFAP2A

## Author contributions

JL and KMSO designed the study. JL, SC, KILC, and CH conducted experiments. JL, KILC, SC, LMSG, and CH analyzed data. JL, SC, and KILC made the figures. JL drafted the manuscript. JL, KILC, and KMSO edited the manuscript. All authors approved the final version of the manuscript. Authorship order among first authors was decided based on JL initiating the project.

## Supplementary Material

Supplemental data

Unedited blot and gel images

Supplemental table 1

Supplemental table 10

Supplemental table 11

Supplemental table 12

Supplemental table 13

Supplemental table 14

Supplemental table 15

Supplemental table 2

Supplemental table 3

Supplemental table 4

Supplemental table 5

Supplemental table 6

Supporting data values

## Version 1 08/28/2025

In-Press Preview

## Version 2 10/08/2025

Electronic publication

## References

[1] Pearce D (2015). Collecting duct principal cell transport processes and their regulation. Clin J Am Soc Nephrol.

[2] Rao R (2019). Intercalated cells of the kidney collecting duct in kidney physiology. Semin Nephrol.

[3] Costantini F, Kopan R (2010). Patterning a complex organ: branching morphogenesis and nephron segmentation in kidney development. Dev Cell.

[4] McMahon AP (2016). Development of the mammalian kidney. Curr Top Dev Biol.

[5] Wang Y (2018). Wnt signaling in kidney development and disease. Prog Mol Biol Transl Sci.

[6] Chambers JM, Wingert RA (2020). Advances in understanding vertebrate nephrogenesis. Tissue Barriers.

[7] Khoshdel Rad N (2020). Cellular and molecular mechanisms of kidney development: from the embryo to the kidney organoid. Front Cell Dev Biol.

[8] van der Ven AT (2018). Novel insights into the pathogenesis of monogenic congenital anomalies of the kidney and urinary tract. J Am Soc Nephrol.

[9] Kagan M (2022). The genetic basis of congenital anomalies of the kidney and urinary tract. Pediatr Nephrol.

[10] Vivante A, Hildebrandt F (2016). Exploring the genetic basis of early-onset chronic kidney disease. Nat Rev Nephrol.

[11] Murugapoopathy V, Gupta IR (2020). A primer on congenital anomalies of the kidneys and urinary tracts (CAKUT). Clin J Am Soc Nephrol.

[12] Westland R (2020). Clinical integration of genome diagnostics for congenital anomalies of the kidney and urinary tract. Clin J Am Soc Nephrol.

[13] Kohl S (2021). Molecular causes of congenital anomalies of the kidney and urinary tract (CAKUT). Mol Cell Pediatr.

[14] Milunsky JM (2008). TFAP2A mutations result in branchio-oculo-facial syndrome. Am J Hum Genet.

[15] Van der Ven AT (2018). Whole-exome sequencing identifies causative mutations in families with congenital anomalies of the kidney and urinary tract. J Am Soc Nephrol.

[16] Mitchell PJ (1991). Transcription factor AP-2 is expressed in neural crest cell lineages during mouse embryogenesis. Genes Dev.

[17] Chazaud C (1996). AP-2.2, a novel gene related to AP-2, is expressed in the forebrain, limbs and face during mouse embryogenesis. Mech Dev.

[18] Moser M (1997). Comparative analysis of AP-2 alpha and AP-2 beta gene expression during murine embryogenesis. Dev Dyn.

[19] Chambers BE (2019). Tfap2a is a novel gatekeeper of nephron differentiation during kidney development. Development.

[20] Miao Z (2021). Single cell regulatory landscape of the mouse kidney highlights cellular differentiation programs and disease targets. Nat Commun.

[21] Sanchez-Ferras O (2021). A coordinated progression of progenitor cell states initiates urinary tract development. Nat Commun.

[23] Milunsky JM (2011). Genotype-phenotype analysis of the branchio-oculo-facial syndrome. Am J Med Genet A.

[24] Lamontagne JO (2022). Transcription factors AP-2α and AP-2β regulate distinct segments of the distal nephron in the mammalian kidney. Nat Commun.

[25] Lindström NO (2021). Spatial transcriptional mapping of the human nephrogenic program. Dev Cell.

[26] Muto Y (2021). Single cell transcriptional and chromatin accessibility profiling redefine cellular heterogeneity in the adult human kidney. Nat Commun.

[27] Ransick A (2019). Single-cell profiling reveals sex, lineage, and regional diversity in the mouse kidney. Dev Cell.

[28] Gerhardt LMS (2023). Lineage tracing and single-nucleus multiomics reveal novel features of adaptive and maladaptive repair after acute kidney injury. J Am Soc Nephrol.

[29] Zhao H (2004). Role of fibroblast growth factor receptors 1 and 2 in the ureteric bud. Dev Biol.

[30] Leiz J (2021). Nuclei isolation from adult mouse kidney for single-nucleus RNA-sequencing. J Vis Exp.

[31] Loganathan R (2020). Extracellular matrix dynamics in tubulogenesis. Cell Signal.

[32] Riga A (2020). New insights into apical-basal polarization in epithelia. Curr Opin Cell Biol.

[33] Papakrivopoulou E (2021). The biological significance and implications of planar cell polarity for nephrology. Front Physiol.

[34] Bowen MA (1995). Cloning, mapping, and characterization of activated leukocyte-cell adhesion molecule (ALCAM), a CD6 ligand. J Exp Med.

[35] Uchida N (1997). The characterization, molecular cloning, and expression of a novel hematopoietic cell antigen from CD34+ human bone marrow cells. Blood.

[36] Chalmers SA (2022). The CD6/ALCAM pathway promotes lupus nephritis via T cell–mediated responses. J Clin Invest.

[37] Cizelsky W (2014). The Wnt/JNK signaling target gene alcam is required for embryonic kidney development. Development.

[38] Karner CM (2009). Wnt9b signaling regulates planar cell polarity and kidney tubule morphogenesis. Nat Genet.

[39] Schunk SJ (2021). WNT-β-catenin signalling - a versatile player in kidney injury and repair. Nat Rev Nephrol.

[40] Lancaster MA (2009). Impaired Wnt-beta-catenin signaling disrupts adult renal homeostasis and leads to cystic kidney ciliopathy. Nat Med.

[41] Walz G, Kim E (2010). Wnt signaling and rejuvenation of the adult kidney. Nephrol Dial Transplant.

[42] Malik SA (2020). The role of Wnt signalling in chronic kidney disease (CKD). Genes (Basel).

[43] Van Otterloo E (2022). AP-2α and AP-2β cooperatively function in the craniofacial surface ectoderm to regulate chromatin and gene expression dynamics during facial development. Elife.

[44] Schorle H (1996). Transcription factor AP-2 essential for cranial closure and craniofacial development. Nature.

[45] Zhang J (1996). Neural tube, skeletal and body wall defects in mice lacking transcription factor AP-2. Nature.

[46] Lienkamp SS (2012). Vertebrate kidney tubules elongate using a planar cell polarity-dependent, rosette-based mechanism of convergent extension. Nat Genet.

[47] Fischer E (2006). Defective planar cell polarity in polycystic kidney disease. Nat Genet.

[48] Jonassen JA (2008). Deletion of IFT20 in the mouse kidney causes misorientation of the mitotic spindle and cystic kidney disease. J Cell Biol.

[49] Saburi S (2008). Loss of Fat4 disrupts PCP signaling and oriented cell division and leads to cystic kidney disease. Nat Genet.

[50] Nishio S (2010). Loss of oriented cell division does not initiate cyst formation. J Am Soc Nephrol.

[51] Simões S de M (2014). Rho GTPase and Shroom direct planar polarized actomyosin contractility during convergent extension. J Cell Biol.

[52] Choe CP (2013). Wnt-dependent epithelial transitions drive pharyngeal pouch formation. Dev Cell.

[53] Jannie KM (2012). ALCAM regulates motility, invasiveness, and adherens junction formation in uveal melanoma cells. PLoS One.

[54] Ohneda O (2001). ALCAM (CD166): its role in hematopoietic and endothelial development. Blood.

[55] Brewer S (2004). Wnt1-Cre-mediated deletion of AP-2alpha causes multiple neural crest-related defects. Dev Biol.

[56] Liao Y (2014). featureCounts: an efficient general purpose program for assigning sequence reads to genomic features. Bioinformatics.

[57] https://www.bioinformatics.babraham.ac.uk/projects/fastqc/.

[58] Livak KJ, Schmittgen TD (2001). Analysis of relative gene expression data using real-time quantitative PCR and the 2(-delta delta C(T)) Method. Methods.

[59] Schneider CA (2012). NIH Image to ImageJ: 25 years of image analysis. Nat Methods.

[60] Hao Y (2021). Integrated analysis of multimodal single-cell data. Cell.

[61] Stuart T (2021). Single-cell chromatin state analysis with Signac. Nat Methods.

[62] Schep AN (2017). chromVAR: inferring transcription-factor-associated accessibility from single-cell epigenomic data. Nat Methods.

[63] Fornes O (2020). JASPAR 2020: update of the open-access database of transcription factor binding profiles. Nucleic Acids Res.

[64] Stuart T (2019). Comprehensive integration of single-cell data. Cell.

[65] Love MI (2014). Moderated estimation of fold change and dispersion for RNA-seq data with DESeq2. Genome Biol.

[66] Wu T (2021). clusterProfiler 4.0: a universal enrichment tool for interpreting omics data. Innovation (Camb).

[67] https://www.R-project.org/.

