# Supplementary material for: TFAP2A orchestrates gene regulatory networks and tubular architecture in kidney outer medullary collecting ducts
Source: JCI Insight. 2025 Aug 28;10(19):e192361. doi: 10.1172/jci.insight.192361 (PMC12513497; doi:10.1172/jci.insight.192361)
Supplement: Supplemental data [file jciinsight-10-192361-s065.pdf]

SUPPLEMENTAL INFORMATION

TFAP2A orchestrates gene regulatory networks and tubular architecture in kidney outer medullary collecting ducts.

Janna Leiz[1,2,3,*], Karen I. López-Cayuqueo[1,*], Shuang Cao[1,2,3], Louisa M.S. Gerhardt[4], Christian Hinze[1,2,3], Kai M. Schmidt-Ott[1,2,3,#]

[1] Department of Nephrology and Hypertension, Hannover Medical School (MHH), Hannover, Germany.

[2] Molecular and Translational Kidney Research, Max-Delbrück Center for Molecular Medicine in the Helmholtz Association, Berlin, Germany

[3]Department of Nephrology and Medical Intensive Care, Charite´ - Universitätsmedizin Berlin, Corporate Member of Freie Universität Berlin and Humboldt-Universität zu Berlin, Berlin, Germany

[4]Fifth Department of Medicine, Faculty of Medicine Mannheim of the University of Heidelberg, University Medical Center Mannheim, Mannheim, Germany.

[*] These authors contributed equally.

[#]**Corresponding author:** Prof. Dr. med. Kai Schmidt-Ott, Department of Nephrology and Hypertension, Hannover Medical School (MHH), 30625 Hannover, Germany. Tel: +49 511 532 6320, Fax: +49 511 552 366, Email: Nephrologie@mh-hannover.de; Schmidt-Ott.Kai@mh-hannover.de

TABLE OF CONTENTS OF SUPPLEMENTAL MATERIAL:

Supplemental Figure S1: Quality control and marker gene expression for murine kidney multiome sequencing dataset.

Supplemental Figure S2: Open chromatin regions around the *Tfap2a* and *Tfap2b* gene bodies and their gene expression in broad kidney cell types in control mice.

Supplemental Figure S3: Body weights, kidney weights, and kidney over body weight ratio of control and *Hoxb7Cre*$^{+}$*;Tfap2a*$^{fl/fl}$ mice at different ages.

Supplemental Figure S4: Outer medullary collecting duct tubule diameter of control and *Hoxb7Cre*$^{+}$*;Tfap2a*$^{fl/fl}$ mice at different ages.

Supplemental Figure S5: Original clustering and quality control for kidney single nucleus RNA-sequencing of control and *Hoxb7Cre*$^{+}$*;Tfap2a*$^{fl/fl}$ mice.

Supplemental Figure S6: Enriched motifs in open chromatin regions associated with differentially expressed genes in outer medullary collecting duct cells.

Supplemental Table S1: Differentially accessible chromatin regions in collecting duct principal cells.

Supplemental Table S2: Enriched motifs in differentially accessible chromatin regions of collecting duct principal cells.

Supplemental Table S3: Transcriptional regulators with differential chromVAR activity in collecting duct principal cells.

Supplemental Table S4: Correlation analysis of differential chromVAR activity and transcription factor gene expression in broad cell types.

Supplemental Table S5: *Tfap2a* motif containing differentially accessible chromatin regions of collecting duct principal cells and their associated genes.

Supplemental Table S6: Enriched biological processes for predicted *Tfap2a* target gene set.

Supplemental Table S7: Plasma creatinine, plasma urea, and blood gas analysis in adult *Hoxb7Cre*$^{+}$*;Tfap2a*$^{fl/fl}$ and control mice under baseline conditions.

Supplemental Table S8: Urinary electrolyte excretion of adult *Hoxb7Cre*$^{+}$*;Tfap2a*$^{fl/fl}$ and control mice under baseline and thirsting conditions.

Supplemental Table S9: Urinary concentration ability of adult *Hoxb7Cre*$^{+}$*;Tfap2a*$^{fl/fl}$ and control mice under baseline and thirsting conditions.

Supplemental Table S10: Single-nucleus RNA-sequencing of *Hoxb7Cre*$^{+}$*;Tfap2a*$^{fl/fl}$ and control mice.

Supplemental Table S11: Expressed genes in broad kidney cell type clusters and subclustered collecting duct principal cells in *Hoxb7Cre*$^{+}$*;Tfap2a*$^{fl/fl}$ versus control mice.

Supplemental Table S12: Gene ontology analysis for deregulated genes in outer medullary collecting duct principal cells.

Supplemental Table S13: In silico ChIP-Seq analysis.

Supplemental Table S14: Expressed genes in whole kidney samples of *Hoxb7Cre*$^{+}$*;Tfap2a*$^{fl/fl}$ versus control mice.

Supplemental Table S15: Gene ontology analysis for deregulated genes in whole kidney bulk RNAseq.

SUPPLEMENTAL FIGURE S1

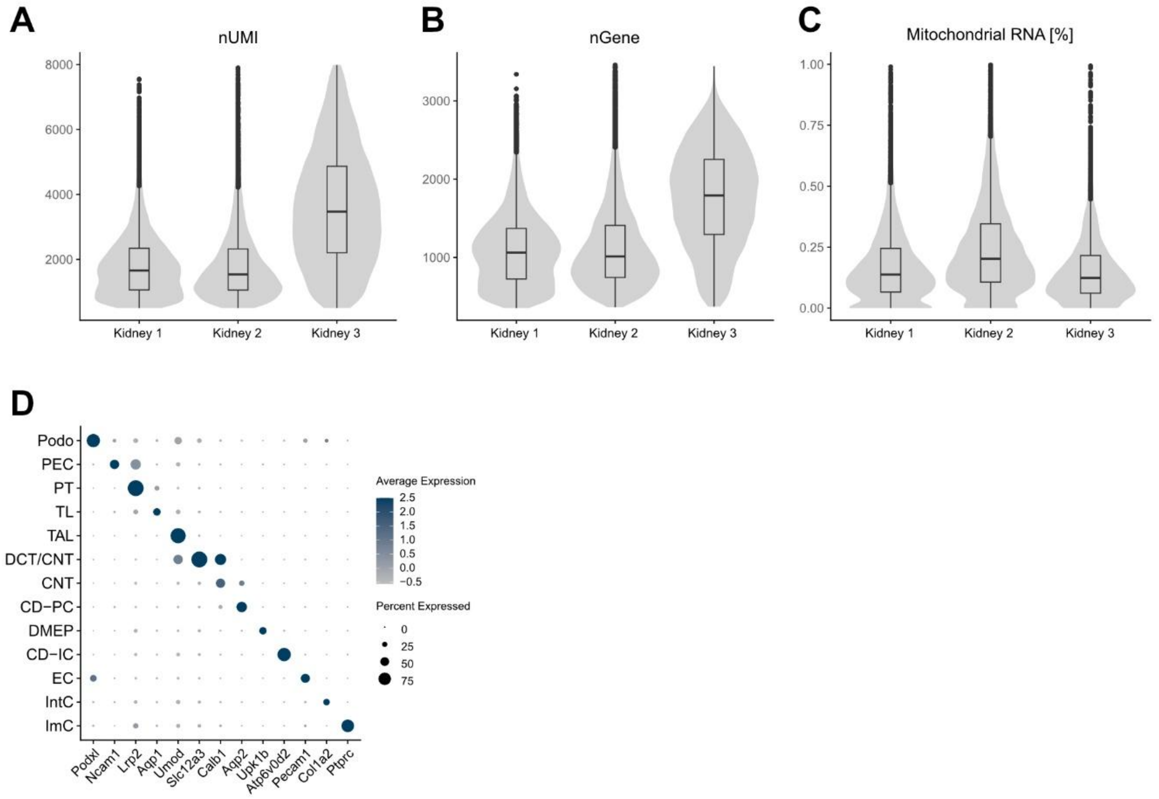

Supplemental Figure S1: Quality control and marker gene expression for murine kidney multiome sequencing dataset.

(A) Distribution of unique molecular identifiers (nUMI), **(B)** number of genes (nGene), and **(C)** percent of mitochondrial RNA detected per nucleus in multiome sequencing data of Ki67cre/ERT2; INTACT (control) mice (n = 3), representing 9,467; 11,374; and 6,961 nuclei, respectively (nuclei with less than 500 or more than 5000 genes and more than 1 % mitochondrial RNA have been excluded). Kidney 1 corresponds to “Control, 4 weeks, rep1”, kidney 2 to “Control, 4 weeks, rep2”, and kidney 3 to “Control, 6 months” from Series GSE209610[1]. **(D)** Dot plot of cell type-specific marker genes for kidney cell types in Ki67cre/ERT2; INTACT mice. Dot color indicates average expression across all cells within a cell type (scaled values), dot size the percentage of cells within a cluster expressing the indicated gene. Podo – podocytes , PEC – parietal epithelial cells, PT – proximal tubule, TL – thin limb, TAL – thick ascending limb, DCT – distal convoluted tubule, CNT – connecting tubule, CD-PC – collecting duct principal cells, CD-IC – collecting duct intercalated cells, DMEP – deep medullary epithelium of the pelvis, EC – endothelial cells, IntC – interstitial cells, ImC – immune cells.

SUPPLEMENTAL FIGURE S2

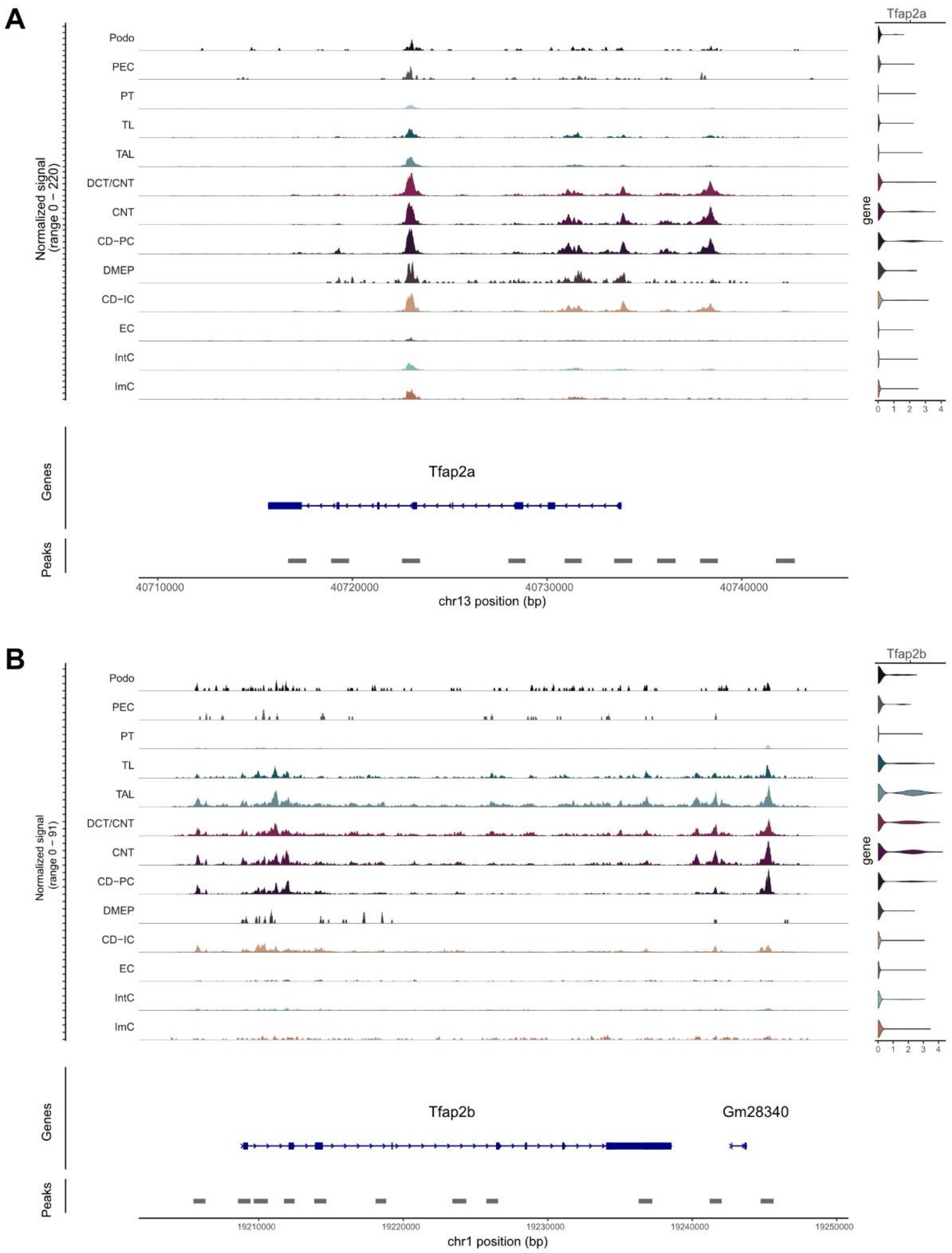

Supplemental Figure S2: Open chromatin regions around the Tfap2a and Tfap2b gene bodies and their gene expression in broad kidney cell types in control mice.

Coverage plots for the **(A)** *Tfap2a* and **(B)** *Tfap2b* gene body (extended 5,000 bp upstream and 10,000 bp downstream), peak locations, and the respective gene expression for all broad cell types identified in N=3 Ki67cre/ERT2; INTACT mice (control mice) from the multiome sequencing data set used in Figure 1 and SupplementalFigure S1. Note high gene accessibility for *Tfap2a*, but low gene accessibility for *Tfap2b* in CD-PC and CD-IC. Podo – podocytes , PEC – parietal epithelial cells, PT – proximal tubule, TL – thin limb, TAL – thick ascending limb, DCT – distal convoluted tubule, CNT – connecting tubule, CD-PC – collecting duct principal cells, CD-IC – collecting duct intercalated cells, DMEP – deep medullary epithelium of the pelvis, EC – endothelial cells, IntC – interstitial cells, ImC – immune cells.

SUPPLEMENTAL FIGURE S3

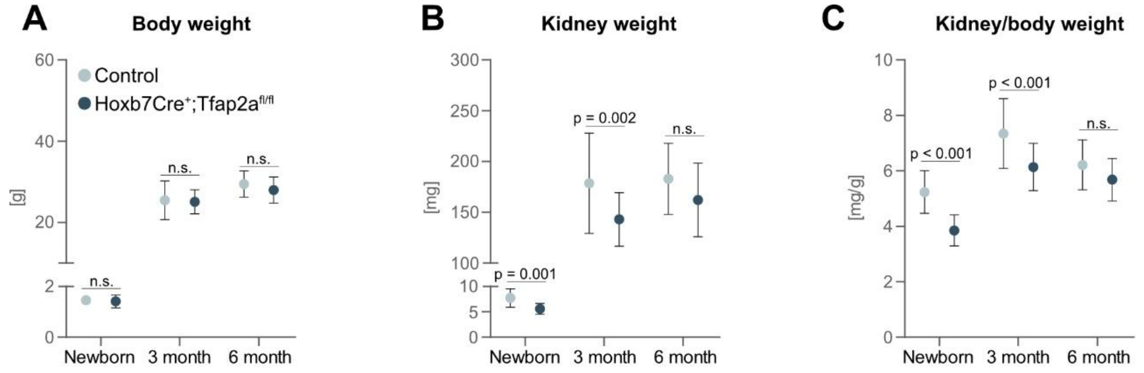

Supplemental Figure S3: Body weights, kidney weights, and kidney over body weight ratio of control and *Hoxb7Cre⁺;Tfap2a*$^{fl/fl}$ mice at different ages.

Comparison of **(A)** body weight, **(B)** kidney weight, and **(C)** kidney weight/body weight ratios of newborn, 3-month, and 6-month-old control and *Hoxb7Cre*$^{+}$*;Tfap2a*$^{fl/fl}$ mice. n ≥ 6 mice per group. Data are expressed as mean ± standard deviation (SD). Statistical significance was determined using a two-tailed t-test, without assuming a consistent SD. n.s. – not significant.

SUPPLEMENTAL FIGURE S4

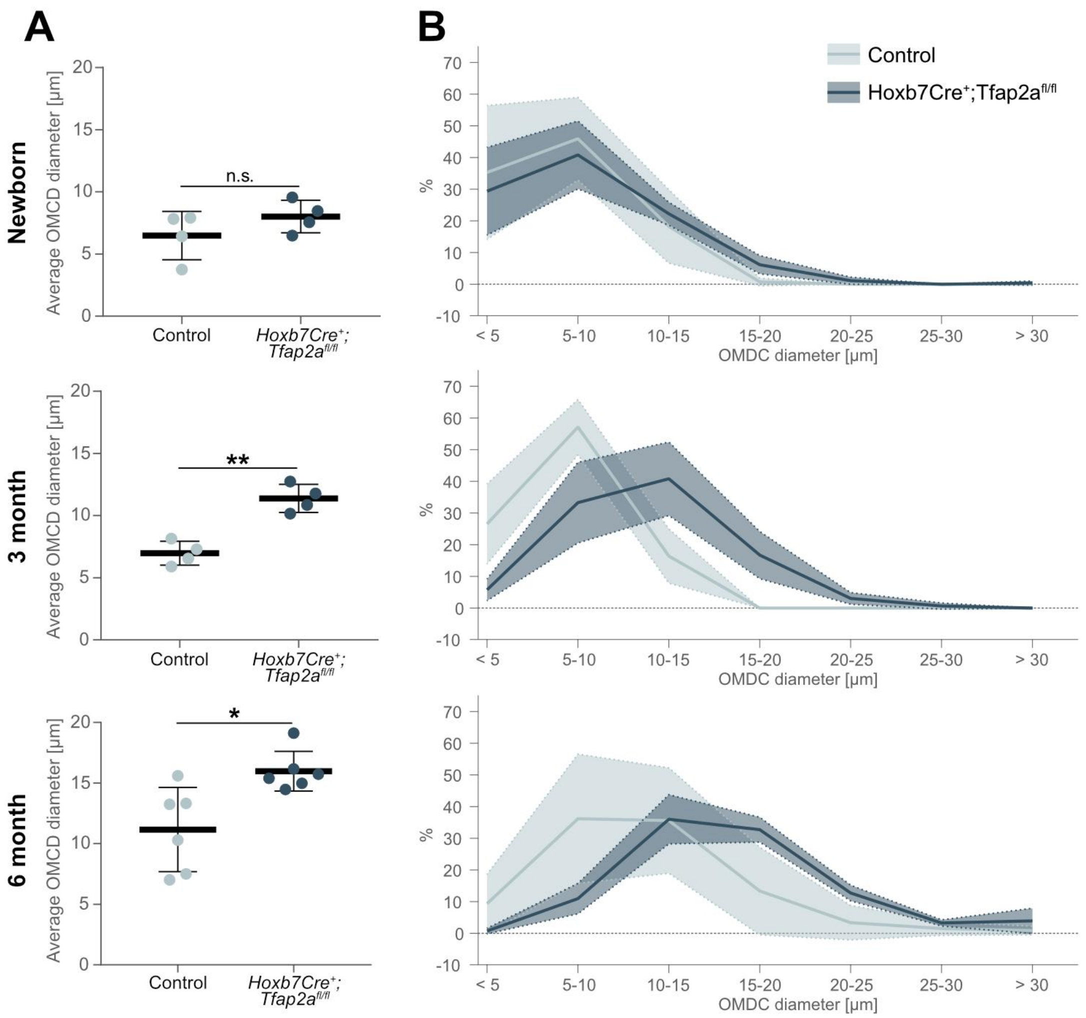

Supplemental Figure S4: Outer medullary collecting duct tubule diameter of control and *Hoxb7Cre^{+};Tfap2a$^{fl/fl}$* mice at different ages.

(A) Measurements of average outer medullary collecting duct (OMCD) diameter for newborn, 3-, and 6-month-old control and *Hoxb7Cre^{+};Tfap2a$^{fl/fl}$* mice. Data are expressed as mean ± standard deviation (SD). n ≥ 4 mice per group. Statistical significance was determined using a two-tailed t-test, without assuming a consistent SD. n.s. – not significant, p < 0.05*, p < 0.01**. **(B)** Percentage of outer medullary collecting ducts within a given diameter range for the same newborn, 3-, and 6-month-old control and *Hoxb7Cre^{+};Tfap2a$^{fl/fl}$* mice as in A. Solid lines represent means, dotted lines and shadowed areas the respective SD.

SUPPLEMENTAL FIGURE S5

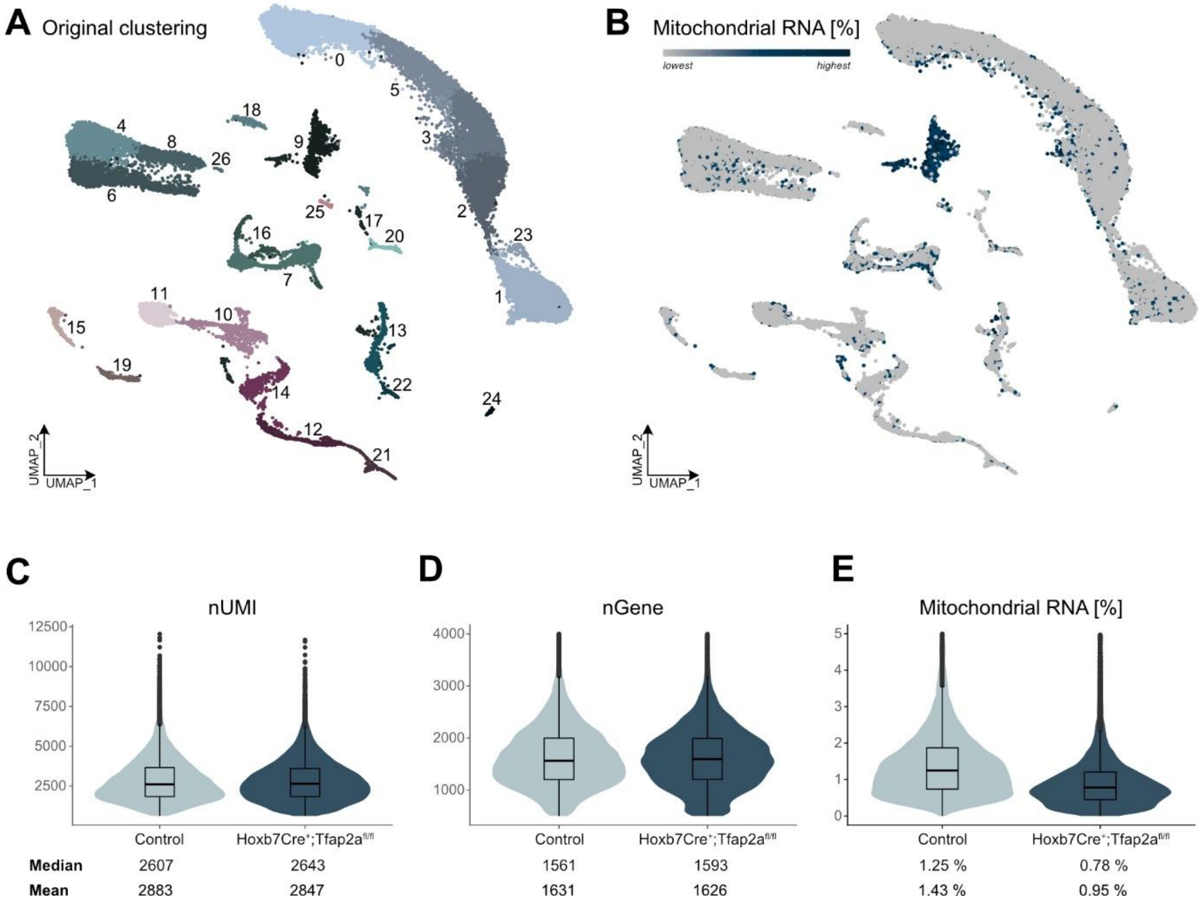

Supplemental Figure S5: Original clustering and quality control for kidney single nucleus RNA-sequencing of control and Hoxb7Cre^{+};Tfap2a$^{fl/fl}$ mice.

(A) Uniform manifold approximation and projection (UMAP) of original clustering for the single-nucleus RNA-sequencing dataset of control and *Hoxb7Cre^{+};Tfap2a$^{fl/fl}$* mice with 27 cluster in total representing 27,341 nuclei (n = 2 mice per group). Nuclei with less than 500 or more than 4000 genes and more than 5 % mitochondrial RNA have been excluded. **(B)** Feature plot displaying expression of mitochondrial RNA. Grey – lowest expression, dark blue – highest expression (5 %). **(C)** Distribution, median, and mean of unique molecular identifiers (nUMI) detected per nucleus in control and *Hoxb7Cre^{+};Tfap2a$^{fl/fl}$* mice. **(D)** Distribution, median, and mean number of genes (nGene) detected per nucleus in control and *Hoxb7Cre^{+};Tfap2a$^{fl/fl}$* mice. **(E)** Distribution, median, and mean percent of mitochondrial RNA reads detected per nucleus in control and *Hoxb7Cre^{+};Tfap2a$^{fl/fl}$* mice.

SUPPLEMENTAL FIGURE S6

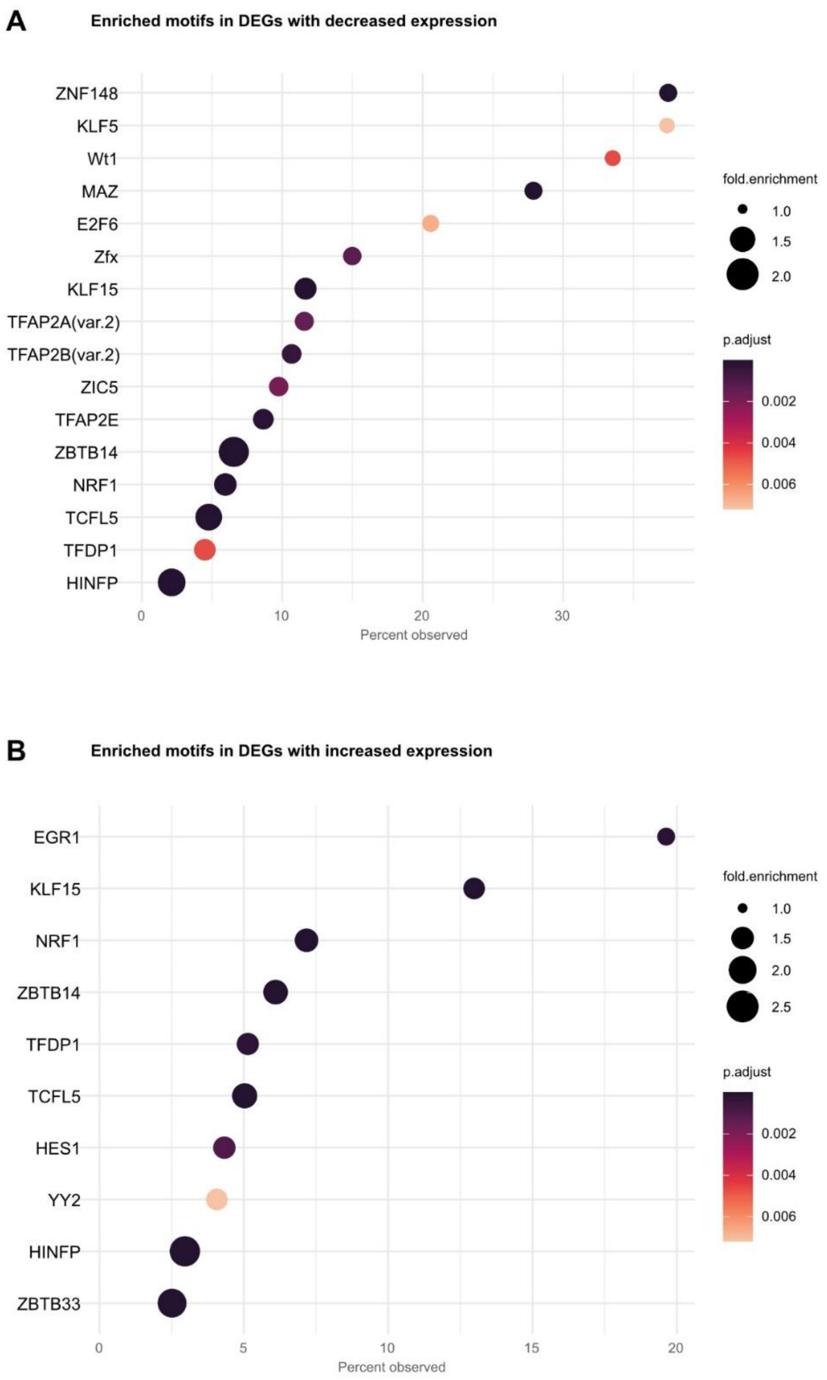

Supplemental Figure S6: Enriched motifs in open chromatin regions associated with differentially expressed genes in outer medullary collecting duct cells.
(A) Enriched motifs in open chromatin regions associated with genes with decreased expression in outer medullary collecting duct cell of *Hoxb7Cre^{+};Tfap2a$^{fl/fl}$* mice when compared to control littermates. **(B)** Enriched motifs in open chromatin regions associated with genes with increased expression in outer medullary collecting duct cell of *Hoxb7Cre^{+};Tfap2a$^{fl/fl}$* mice when compared to control littermates. Motifs are sorted by observed frequency (= percent of open chromatin regions containing motif). Colors represent the adjusted p-value for motif enrichment; dot size indicates the fold enrichment in comparison to the background data set.

SUPPLEMENTAL TABLE S1: Differentially accessible chromatin regions in collecting duct principal cells.

List of differentially accessible regions (DAR) significantly enriched (adjusted p value < 0.01) with an average log 2 foldchange > 2 in collecting duct principal cells when compared to other kidney cell types (n = 1,933). **This table is provided as an Excel file.**

SUPPLEMENTAL TABLE S2: Enriched motifs in differentially accessible chromatin regions of collecting duct principal cells.

Enriched transcription factor motifs (p.adjust<0.05, foldchange > 1.5) within collecting duct principal cell DAR as identified by Signac. **This table is provided as an Excel file.**

SUPPLEMENTAL TABLE S3: Differentially activated motifs in collecting duct principal cells.

List of differentially activated motifs and their associated genes as calculated with chromVAR (p < 0.01). **This table is provided as an Excel file.**

SUPPLEMENTAL TABLE S4: Correlation analysis of differential transcription factor chromVAR activity and gene expression in broad cell types.

Correlation analysis for all TF-motif combinations in all cell types. Only cell types with significant differential expression or chromVAR activity are included in the list (p < 0.05). **This table is provided as an Excel file.**

SUPPLEMENTAL TABLE S5: Tfap2a motif containing differentially accessible chromatin regions of collecting duct principal cells and their associated genes.

Differentially accessible open chromatin regions in collecting duct principal cells were filtered for the presence of *Tfap2a* motifs (n = 625). **This table is provided as an Excel file.**

SUPPLEMENTAL TABLE S6: Enriched biological processes for predicted Tfap2a target gene set.

Gene ontology analysis for predited Tfap2a target gene set performed with clusterProfiler (pAdjustMethod = "fdr", pvalueCutoff = 0.05, qvalueCutoff = 0.05, minGSSize = 2). **This table is provided as an Excel file.**

SUPPLEMENTAL TABLE S7

Plasma creatinine, plasma urea, and blood gas analysis in adult *Hoxb7Cre⁺;Tfap2a^fl/fl^* and control mice under baseline conditions.

	**Control (n ≥ 6)**		***Hoxb7Cre^{+};Tfap2a$^{fl/fl}$* (n ≥ 4)**		
	Mean	SD	Mean	SD	p-value

**Plasma creatinine and urea (baseline)**						
Creatinine	mg/dl	0.0963	0.0343	0.084	0.0502	0.5710
Urea	mg/dl	48.57	11.08	49.88	7.335	0.8315
**Blood gas analysis (baseline)**						
Na^+	mmol/l	145	1.07	146	1.22	0.223
K^+	mmol/l	4.4	0.48	5.6	0.38	0.007
iCa	mmol/l	1.35	0.05	1.36	0.06	0.722
Glu	mg/dl	212	26.98	183	41.87	0.332
Hct	%PCV	44	0.75	45	1.22	0.438
Hgb	g/dl	15.1	0.25	15.3	0.41	0.501
pH		7.25	0.1	7.2	0.12	0.6
pCO_2	mmHg	55.9	16.77	65.3	17.85	0.485
pO_2	mmHg	41.8	23.55	24.3	6.53	0.166
TCO_2	mmol/l	25.5	3.30	26.5	2.5	0.624
HCO_3	mmol/l	23.7	3.1	24.55	2.15	0.66
BE	mmol/l	-3.5	3.45	-3.5	2.29	1
sO_2	%	56	33	33	20	0.246

Creatinine and urea were determined in blood plasma samples of 3-month-old mice. Blood gas parameter were determined in fresh whole blood samples of 2–3-month-old mice using the iStat as described in the methods section. Values represent mean ± standard deviation (SD). Statistical significance was determined using a two-tailed t-test, without assuming a consistent SD.

Na^+ – Sodium, K^+ – potassium, iCa – ionized calcium, Glu – glucose, Hct – hematocrit, Hgb – hemoglobin, pH – blood pH, pCO_2 – partial pressure of carbon dioxide, pO_2 – partial pressure of oxygen, TCO_2 – total amount of carbon dioxide, HCO_3 – hydrogen carbonate, BE – base excess, sO_2 – oxygen saturation.

SUPPLEMENTAL TABLE S8

Urinary electrolyte excretion of adult *Hoxb7Cre⁺;Tfap2a^fl/fl* and control mice under baseline and thirsting conditions.

		**Control (n ≥ 11)**		***Hoxb7Cre*$^+$*;Tfap2a*$^{fl/fl}$ (n ≥ 13)**		
		Mean	SD	Mean	SD	p-value
**Baseline**						
Creatinine	mg/dl	21.31	6.417	20.46	4.139	0.6960

Urea	mg/dl	4105	1140	3508	850.3	0.1491
Sodium	mmol/l	87.83	23.86	83.2	19.39	0.5973
Potassium	mmol/l	122.3	25.4	117.3	29.97	0.6596
Chloride	mmol/l	84.81	21.85	77.44	18.04	0.3652
Calcium	mg/dl	7.44	4.019	6.001	1.631	0.2505
Magnesium	mg/dl	28.63	7.025	23.83	6.464	0.0875
Phosphate	mg/dl	208	80.24	217.3	73.2	0.7646
**Thirst (24h)**						
Creatinine	mg/dl	27.315	11.3	31.445	14.72	0.4543
Urea	mg/dl	4184	1330	4761	1921	0.4045
Sodium	mmol/l	103.049	35.393	106.739	40.403	0.8179
Potassium	mmol/l	131.645	32.392	135.644	31.5	0.7693
Chloride	mmol/l	94.704	32.26	98.417	37.711	0.8016
Calcium	mg/dl	19.43	8.988	13.78	6.594	0.1122
Magnesium	mg/dl	27.857	13.282	26.362	13.621	0.8000
Phosphate	mg/dl	216.206	86.506	240.146	103.467	0.5519

3-month-old *Hoxb7Cre*$^{+}$*;Tfap2a*$^{fl/fl}$ mice and littermate controls were kept in metabolic cages for 24 h under water ad libitum (baseline) or without water (thirst). Values represent mean ± standard deviation (SD). Statistical significance was determined using a two-tailed t-test, without assuming a consistent SD.

SUPPLEMENTAL TABLE S9

Urinary concentration ability of adult *Hoxb7Cre^{+};Tfap2a$^{fl/fl}$* and control mice under baseline and thirsting conditions.

		n	Mean	SD	p-value
**Baseline (24h)**					
Drinking volume	Control	14	0.058	0.0387	0.5645
[ml/g bw/24h]	Hoxb7Cre^{+};Tfap2a$^{fl/fl}$	13	0.067	0.0378	
Urinary output	Control	14	61.87	29.14	0.8161
[µl/g bw/24h]	Hoxb7Cre^{+};Tfap2a$^{fl/fl}$	13	64.47	26.11	
Osmolality	Control	14	1275	263.8	0.3724
[mosmol/kg]	Hoxb7Cre^{+};Tfap2a$^{fl/fl}$	13	1169	313.8	
Body weight loss	Control	14	13.12	5.013	0.9434
[%]	Hoxb7Cre^{+};Tfap2a$^{fl/fl}$	13	13.27	5.376	
**Thirst (24h)**					
Urinary output	Control	11	44.56	18.78	0.1746
[µl/g bw/24h]	Hoxb7Cre^{+};Tfap2a$^{fl/fl}$	14	33.59	18.29	
Osmolality	Control	11	1362	411.3	0.6340
[mosmol/kg]	Hoxb7Cre^{+};Tfap2a$^{fl/fl}$	14	1453	500.0	
Body weight loss	Control	11	20.04	4.1832	0.9762
[%]	Hoxb7Cre^{+};Tfap2a$^{fl/fl}$	14	19.98	4.9534	

Daily drinking volume, urinary output, osmolality, and body weight loss were determined in 3-month-old Hoxb7Cre^{+};Tfap2a$^{fl/fl}$ mice and littermate controls. Mice were kept in metabolic cages for 24 h with water ad libitum (baseline) or without water (thirst). Urinary output and drinking volume were normalized to body weight (bw). Values represent mean ± standard deviation (SD). Statistical significance was determined using a two-tailed t-test, without assuming a consistent SD.

SUPPLEMENTAL TABLE S10: Single-nucleus RNA-sequencing of *Hoxb7Cre⁺;Tfap2a^fl/fl^* and control mice.

Sample information and cell type abundances for *Hoxb7Cre+;Tfap2a*$^{fl/fl}$ and control mice. **This table is provided as an Excel file.**

SUPPLEMENTAL TABLE S11: Expressed genes in broad kidney cell type clusters and subclustered collecting duct principal cells in *Hoxb7Cre*$^{+}$*;Tfap2a*$^{fl/fl}$ versus control mice.

Expressed genes calculated with "FindMarkers" function in Seurat for each cell type (min.pct = 0.05 for broad cell types, min.pct = 0.01 for CD-PC subcluster (only tests genes that are detected in a minimum fraction of 5 % or 1 % of cells in either knockout or control samples), logfc.threshold = 0.25 (limits testing to genes which show, on average, at least 0.25-fold difference (log-scale) between knockout and control), test used to identify differentially expressed genes between knockout and control = Wilcoxon Rank Sum test). Thresholds to be considered differentially expressed genes: p-value < 0.05; foldchange ± 1.3. **This table is provided as an Excel file.**

SUPPLEMENTAL TABLE S12: Gene ontology analysis for deregulated genes in outer medullary collecting duct principal cells.

Gene ontology analysis for genes deregulated in *Tfap2a*-deficent outer medullary collecting duct cells performed with clusterProfiler (pAdjustMethod = "fdr", pvalueCutoff = 0.05, qvalueCutoff = 0.05, minGSSize = 2). **This table is provided as an Excel file.**

SUPPLEMENTAL TABLE S13: In silico ChIP-Seq analysis.

Open chromatin regions associated with differentially expressed genes were filtered for the presence of at least one Tfap2a motif (regions = 1386; individual genes = 218). **This table is provided as an Excel file.**

SUPPLEMENTAL TABLE S14: Expressed genes in whole kidney samples of *Hoxb7Cre*$^{+}$*;Tfap2a*$^{fl/fl}$ versus control mice.

Expressed genes as identified in bulk RNA-sequencing (RNAseq) from whole kidney samples using DESeq2: Whole kidney samples of 4 control versus 4 knockout (*Hoxb7Cre:Tfap2a*$^{fl/fl}$) animals

Wald test for differential expression testing; Benjamini and Hochberg method for multiple testing correction. Thresholds set to be considered differentially expressed genes: Gene count (baseMean) ≥ 5, P-value < 0.05, Adjusted P-value < 0.05, Foldchange ± 1.3. **This table is provided as an Excel file.**

SUPPLEMENTAL TABLE S15: Gene ontology analysis for deregulated genes in whole kidney bulk RNAseq.

Gene ontology analysis for genes deregulated in *Hoxb7Cre*$^{+}$*;Tfap2a*$^{fl/fl}$ whole kidney bulk RNAseq samples. Enriched pathways were analyzed using the enrichGO() function, enriched biological pathways were determined separately for genes up- and downregulated in the respective dataset. Adjustments of p-values were calculated using the false discovery rate. Terms with a p- and q-value cutoff < 0.05 were considered significant. **This table is provided as an Excel file.**

REFERENCES

(1) Gerhardt, L. M. S.; Koppitch, K.; van Gestel, J.; Guo, J.; Cho, S.; Wu, H.; Kirita, Y.; Humphreys, B. D.; McMahon, A. P. Lineage Tracing and Single-Nucleus Multiomics Reveal Novel Features of Adaptive and Maladaptive Repair after Acute Kidney Injury. *J Am Soc Nephrol* **2023**, *34* (4), 554–571. https://doi.org/10.1681/ASN.0000000000000057.
