# Supplementary figures and images for: TFAP2A orchestrates gene regulatory networks and tubular architecture in kidney outer medullary collecting ducts
Source: JCI Insight. 2025 Aug 28;10(19):e192361. doi: 10.1172/jci.insight.192361 (PMC12513497; doi:10.1172/jci.insight.192361)

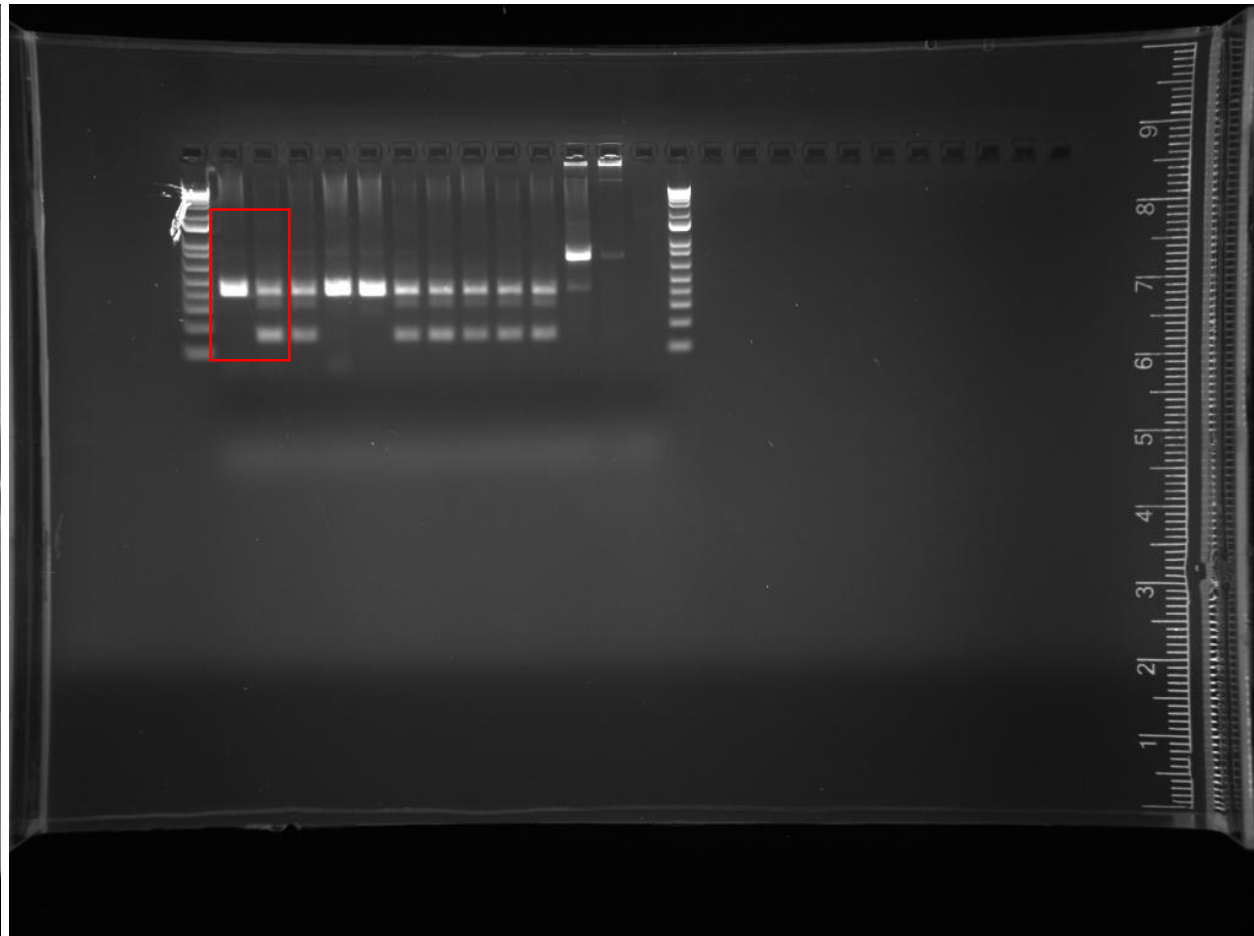

Supplement: Unedited blot and gel images [file jciinsight-10-192361-s066.pdf]
